# Empowering individual trait prediction using interactions for precision medicine

**DOI:** 10.1186/s12859-021-04011-z

**Published:** 2021-02-18

**Authors:** Damian Gola, Inke R. König

**Affiliations:** Institut für Medizinische Biometrie und Statistik, Universität zu Lübeck, Universitätsklinikum Schleswig-Holstein, Campus Lübeck, Lübeck, Germany

**Keywords:** Prediction, Machine learning, Interactions, Classification

## Abstract

**Background:**

One component of precision medicine is to construct prediction models with their predicitve ability as high as possible, e.g. to enable individual risk prediction. In genetic epidemiology, complex diseases like coronary artery disease, rheumatoid arthritis, and type 2 diabetes, have a polygenic basis and a common assumption is that biological and genetic features affect the outcome under consideration via interactions. In the case of omics data, the use of standard approaches such as generalized linear models may be suboptimal and machine learning methods are appealing to make individual predictions. However, most of these algorithms focus mostly on main or marginal effects of the single features in a dataset. On the other hand, the detection of interacting features is an active area of research in the realm of genetic epidemiology. One big class of algorithms to detect interacting features is based on the multifactor dimensionality reduction (MDR). Here, we further develop the model-based MDR (MB-MDR), a powerful extension of the original MDR algorithm, to enable interaction empowered individual prediction.

**Results:**

Using a comprehensive simulation study we show that our new algorithm (median AUC: 0.66) can use information hidden in interactions and outperforms two other state-of-the-art algorithms, namely the Random Forest (median AUC: 0.54) and Elastic Net (median AUC: 0.50), if interactions are present in a scenario of two pairs of two features having small effects. The performance of these algorithms is comparable if no interactions are present. Further, we show that our new algorithm is applicable to real data by comparing the performance of the three algorithms on a dataset of rheumatoid arthritis cases and healthy controls. As our new algorithm is not only applicable to biological/genetic data but to all datasets with discrete features, it may have practical implications in other research fields where interactions between features have to be considered as well, and we made our method available as an R package (https://github.com/imbs-hl/MBMDRClassifieR).

**Conclusions:**

The explicit use of interactions between features can improve the prediction performance and thus should be included in further attempts to move precision medicine forward.

## Background

The concept of precision medicine is meant to improve many aspects of health and healthcare. It promises a new level of disease treatment and prevention for complex diseases like coronary artery disease, rheumatoid arthritis, and type 2 diabetes, by taking into account individual variability in genes, environment, and lifestyle. In the long-term, healthcare professionals and researchers will be able to predict more accurately which treatment and prevention strategies for a particular disease will work in which groups of patients. To achieve this level, precision medicine can be viewed as a continuous process of data preprocessing/data mining (track 1), construction of diagnostic/prognostic models (track 2) and prediction of treatment response/disease progression (track 3) [1]. Whereas track 1 focuses on the identification of important observed and latent variables, tracks 2 and 3 require models with highly accurate predictions about disease status, prognosis or progression of a disease of a single individual [2–6]. Explained with (generalized) linear models as an example, based on the estimation of and inference on regression coefficients, tracks 2 and 3 aim at constructing models with their predictive ability as high as possible, measured in terms of some performance, e.g. the area under the receiver operating characteristic curve (AUC). In genetic epidemiology, simple Mendelian diseases, such as cystic fibrosis and hereditary breast and ovarian cancer, allow for relatively straightforward predictions. However, more complex diseases like those mentioned above, involve complex molecular mechanisms and thus have a polygenic basis [7]. It is a common assumption that these biological/genetic features, such as proteins and the underlying genetic variations, also are acting via interactions either with each other [8–11] or with environmental features [12]. To make it even more complicated, features may affect the outcome under consideration only via interactions. Thus, the interacting features do not have an effect on their own. An example of such a constellation is the effect of a variant in the *MDR1* gene together with exposure to pesticides on Parkinson’s disease [13].

The use of standard approaches such as generalized linear models is suboptimal in these cases because of the algorithm instabilities when modeling many variables and their interactions or requirements of large sample sizes [14]. Thus, regularized generalized linear models [15, 16] or machine learning methods, e.g. Random Forest [17], are appealing to make individual predictions based on many variables. They differ in the details, but most of them share one important property: they focus mostly on main or marginal effects of the single features in a dataset. For example, in Random Forest, at each node, the single best feature and its best split point are selected [18]. This may lead to ignoring features without any or only small main effects, although Wright et al. [19] have shown that using enough single trees can compensate for this issue. Likewise, regularized regression models are usually specified using main effect terms only, and interaction terms have to be included explicitly as new features [20]. These common limitations may limit the prediction performance of models based on currently used algorithms if features have an effect on the outcome only via interactions. Further, these algorithms are at opposite corners in terms of performance and interpretability. Whereas the prediction performance of Random Forests is generally at the highest levels, their interpretability is somewhat limited, especially, if there are thousands of trees in a single forest. The very opposite is true for regularized regression models. Their prediction performance may be limited by the fact that the models are made up of simple additive effects of the underlying features, however, they thus offer good interpretability in general, which might be of great interest in precision medicine.

On the other hand, there has been much research on the detection of interacting features in the realm of genetic epidemiology [21]. One big class of algorithms to detect interacting features are the multifactor dimensionality reduction (MDR)-based algorithms based on the original idea by Ritchie et al. [22]. The basic idea of all MDR-based algorithms is to reduce the dimensionality of simultaneously analyzed features by pooling combinations of feature levels (*cells*) in high risk ($H$) and low risk ($L$) groups, resulting in a single best combination (*MDR model*) of $d$ features. The original MDR algorithm has several drawbacks and limitations, thus a large number of modifications and extensions were proposed in recent years. A comprehensive review of the original MDR algorithm and its modifications and extensions is given by Gola et al. [23]. However, these algorithms aim at identifying interacting features but do not allow for individual predictions.

In this work we show how to extend the model-based MDR (MB-MDR), a powerful MDR-based algorithm to detect interacting features first described by Calle et al. [24], to enable interaction empowered individual prediction while maintaining interpretability of the prediction models. We do this, inspired by the methodology of the Random Forest algorithm, by considering each combination of features as a classification model in itself and by aggregating an optimal number of these models. The optimal number is found by internal cross-validation. Here, we focus on presenting our new algorithm and its comparison with the performance of Random Forest and Elastic Net in a comprehensive simulation study. For illustrational purposes, we also apply all three algorithms to a dataset by the North American Rheumatoid Arthritis Consortium (NARAC).

## Results

### Simulation study

A simulation study was performed to compare our proposed algorithm with two state-of-the-art prediction algorithms, the Random Forest [17] and the Elastic Net [31], a generalization of the LASSO [16] and ridge regression [15], for classification tasks. As implementations we utilized the R (version 3.3.1) [32] packages ranger (version 0.8.1.300) [33] and glmnet (version 2.0-5) [20]. We considered eight scenarios to investigate the performance of the three algorithms given different underlying effect structures. The scenarios start with very simple effect structures and gradually become more complex. In each scenario different simulation parameter combinations were considered. For each scenario and combination of simulation parameters 50 datasets $D$ were created as replicates. In each replication we independently simulated $q=100$ SNPs in total, and of those different numbers of SNPs or combinations of SNPs are used as effect feature components.

#### Benchmark

For the benchmarking regarding the AUC of the three algorithms, we used the mlr framework (version 2.12) [35]. Each dataset $D$ was split into datasets ${D}_{1}$ and ${D}_{2}$ of the same size. Tuning was performed with fivefold cross-validation on ${D}_{1}$ using the R package mlrMBO (version 1.1.0) [36] for 100 iterations with ranger (ntrees: 500, mtry: square root of the number of tuning hyperparameters) as the surrogate learner. After tuning, a prediction model with the tuned parameters was built on ${D}_{1}$ and the prediction performance was calculated on ${D}_{2}$ for each replicate.

#### Scenarios 1 and 2: only main effects

**Fig. 1:**
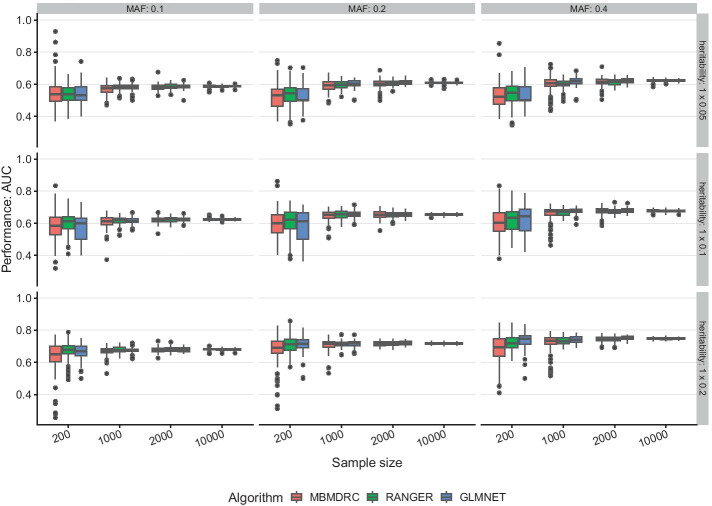
Performance in simulation scenario 1. Performance of the algorithms MBMDRC, RANGER, and GLMNET measured as AUC over 50 replicates in sample sizes 200, 1000, 2000, and 10,000 in scenario 1: one SNP with main effect (MAF 0.1, 0.2, or 0.4 and heritability 0.05, 0.1, 0.2), 99 SNPs without any effect

**Fig. 2:**
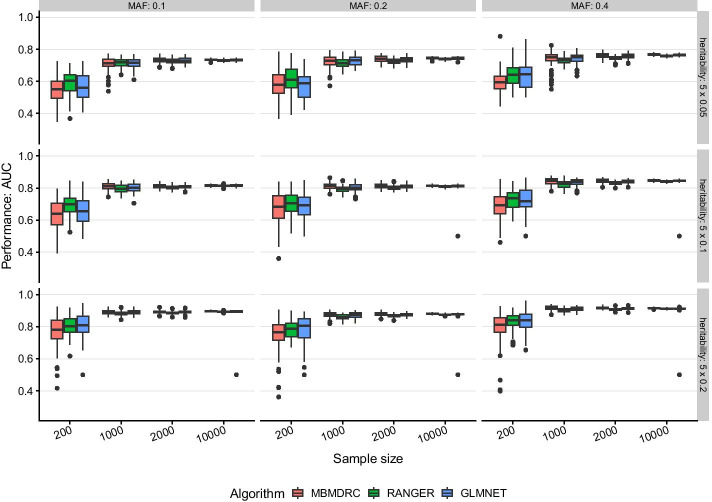
Performance in simulation scenario 2. Performance of the algorithms MBMDRC, RANGER, and GLMNET measured as AUC over 50 replicates in sample sizes 200, 1000, 2000, and 10,000 in scenario 2: five SNPs with main effects (MAF 0.1, 0.2, or 0.4 and heritability 0.05, 0.1, 0.2), 95 SNPs without any effect

In scenarios 1 (one effect SNP) and 2 (five effect SNPs) with only main effects simulated, all algorithms achieve similar performances. All algorithms show the greatest variability of performance across the 50 simulated data sets as indicated by the height of the box and the dots in Figs. 1 and 2 for the lowest sample sizes of 200, i.e. 100 for tuning and training and 100 for performance estimation. With increasing sample size, the median performance increases and the variability of performance decreases for all algorithms. The heritability has the greatest impact on prediction performance. For example, the AUC increases from about 0.62 to 0.68 to 0.75 for heritabilities 0.05, 0.1 and 0.2 in scenario 1 and 10,000 samples. Comparing these two scenarios shows that the performance is dependent on the number of SNP combinations, i.e. in these scenarios the number of SNPs with main effect. This is expected, as the total heritability increases with the number of SNPs.

#### Scenario 3: one pair of two interacting SNPs

**Fig. 3:**
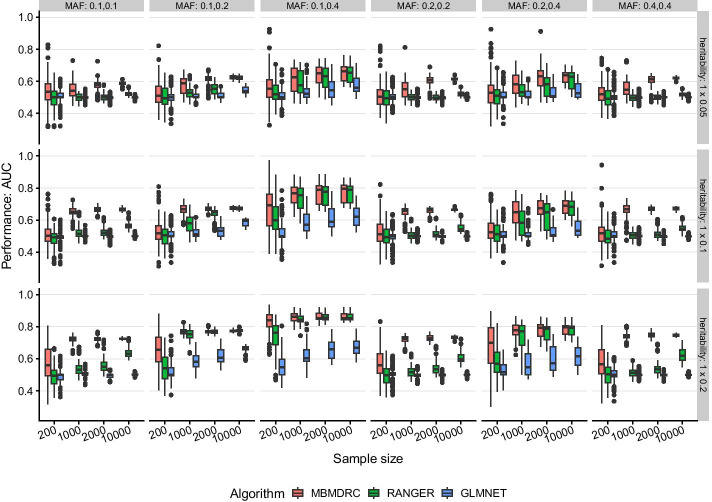
Performance in simulation scenario 3. Performance of the algorithms MBMDRC, RANGER, and GLMNET measured as AUC over 50 replicates in sample sizes 200, 1000, 2000, and 10,000 in scenario 3: one pair of interacting SNPs without marginal effects (MAF 0.1, 0.2, or 0.4 and heritability 0.05, 0.1, 0.2), 98 SNPs without any effect

Simulating only one interaction effect as in scenario 3, MBMDRC models have the highest median prediction performance for all sample sizes as shown in Fig. 3. In this scenario, the ranger and glmnet models do not achieve a median performance greater than 0.55, if the interacting SNPs have the same MAF. Interestingly, models can improve their median performance at the cost of increased variability, if the SNPs have different MAFs. This effect is most evident for ranger models, but also observable for the other two algorithms.

#### Scenario 7: three pairs of interacting SNPs and three SNPs with main effects

**Fig. 4:**
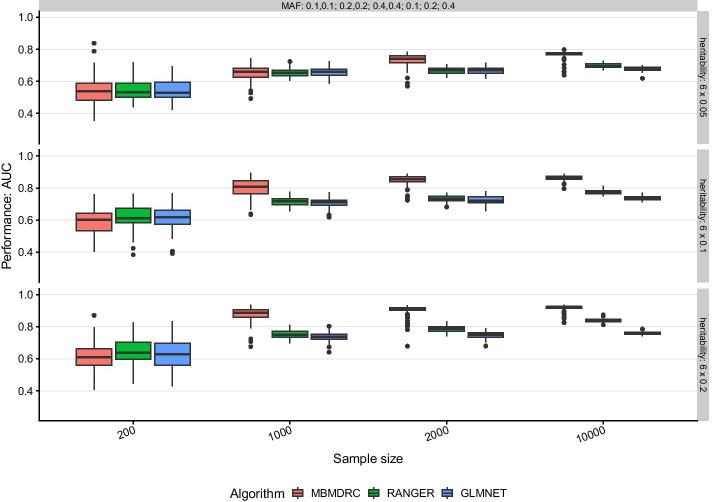
Performance in simulation scenario 7. Performance of the algorithms MBMDRC, RANGER, and GLMNET measured as AUC over 50 replicates in sample sizes 200, 1000, 2000, and 10,000 in scenario 7: three pairs of interacting SNPs without marginal effects and three SNPs with marginal effects only (MAF 0.1, 0.2, or 0.4 and heritability 0.05, 0.1, 0.2), 91 SNPs without any effect

For scenarios with both main and interaction effects, MBMDRC models dominate the other two algorithms for sample sizes greater than 200 (see Fig. 4 for scenario 7). However, ranger models can reach similar or even better performances if the interacting SNPs have different MAFs. For example, for MAFs 0.1 and 0.4, heritability greater or equal 0.1 and sample size greater than 1000, ranger models achieve a better performance than the MBMDRC models on the median, although the variability is slightly increased. The glmnet models do not use the interaction information, thus their performance is just based on the available main effects and the maximum median performances remain at about the same level between 0.68 and 0.80 as in scenario 1.

#### Scenario 8: one interaction of three SNPs and three SNPs with main effects

**Fig. 5:**
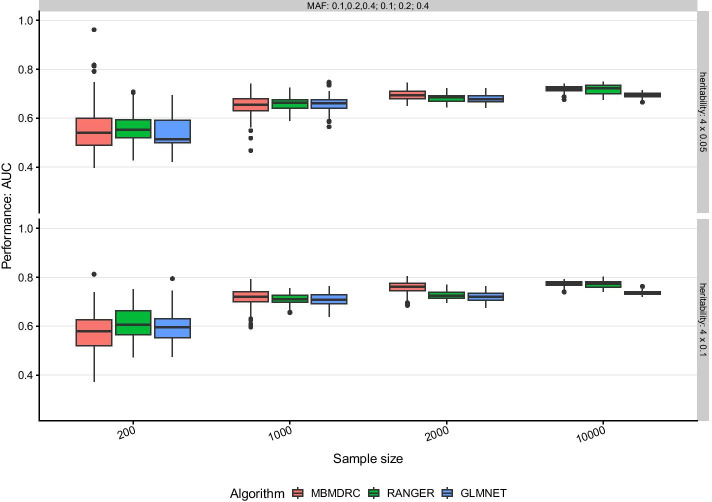
Performance in simulation scenario 8. Performance of the algorithms MBMDRC, RANGER, and GLMNET measured as AUC over 50 replicates in sample sizes 200, 1000, 2000, and 10,000 in scenario 8: three interacting SNPs without marginal effects and three SNPs with marginal effects only (MAF 0.1, 0.2, or 0.4 and heritability 0.05, 0.1, 0.2), 94 SNPs without any effect

In scenario 8, the interaction of three SNPs was simulated, which is an interaction of higher order than considered by the MB-MDR. Still, MBMDRC models achieve at least a similar performance as the glmnet and ranger models (see Fig. 5). Even though the MBMDRC models should be based mostly on the three additional single SNPs with marginal effects, the median performance for sample size 2000 is slightly better than those of ranger and glmnet. For sample size 10,000, ranger can achieve similar median performance but with higher variability. The glmnet models are limited by the information based on the three main effects and their performance is comparable to those of scenario 7.

#### Other scenarios

The further scenarios 4, 5, and 6 confirm the relationships described so far and the corresponding Additional files 1–3: Figures 1–3 as well as detailed result tables also for the already described scenarios (see Additional files 4–11: Tables 1–8) can be found as additional files. ROC curves for sample size 100,000 in each scenario are shown in Additional files 13–20: Figures 4–11.

### Real data

**Fig. 6:**
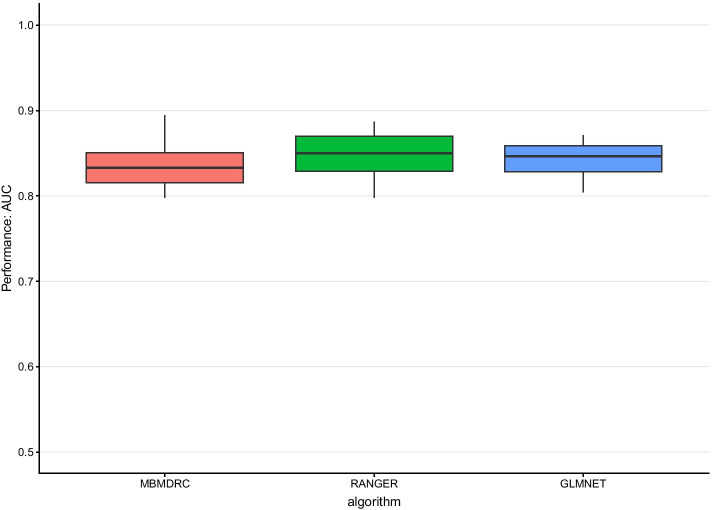
Performance in the NARAC dataset. Performance of the algorithms MBMDRC, RANGER, and GLMNET measured as AUC in tenfold cross validation in the NARAC dataset of 868 cases and 1194 controls with 18,263 SNPs from the HLA region on chromosome 6

Application of the three algorithms to the rheumatoid arthritis dataset yields no relevant differences regarding their median performance. Here, the glmnet models have a median AUC of 0.86, the ranger models of 0.85 and the MBMDRC models of 0.83, comparable to the simulation results of scenario 2, i.e. multiple SNPs with main effects but no interactions, MAF = 0.4, ${h}^{2}=0.1$, and sample size between 1000 and 2000. The corresponding box plot is shown in Fig. 6.

**Fig. 7:**
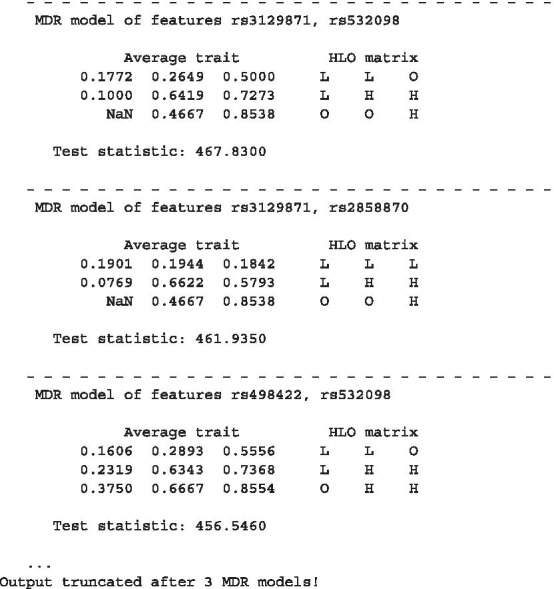
First 3 MDR models of an MBMDRC model. The average trait, i.e. for the NARAC dataset the fraction of cases, and the respective cell classifications are shown. The data is taken from one randomly selected MBMDRC model of the benchmark on the NARAC dataset of 868 cases and 1194 controls with 18,263 SNPs from the HLA region on chromosome 6. On the left side the average trait in each cell, i.e. for the NARAC dataset the fraction of cases, and on the right side, the respective classifications are displayed. In this case, the average traits could be interpreted as estimates of the respective genotype penetrances

In contrast to both other algorithms, the MBMDRC models allow insight into the underlying effect structure of the features. In Fig. 7, the three MDR models with the highest test statistics taken from a random MBMDRC model are shown. On the left side the average trait in each cell, i.e. for the NARAC dataset the fraction of cases, and on the right side, the respective classifications are displayed. In this case, the average traits could be interpreted as estimates of the respective genotype penetrances. For example, the HLO matrix of the MDR model of the features rs498422 and rs532098 can be interpreted as follows:

1. Samples must have at least one minor allele at both SNPs to have a higher disease risk.
2. Having two minor alleles at one SNP but two major alleles at the other SNP results in no significant risk increase or decrease.
3. All other genotype combinations decrease disease risk.

Comparison of the runtimes shows that the ranger implementation is the fastest of all three. Specifically, the mean runtime on Intel® Xeon® E5-2680 CPUs at 2.70 GHz of one outer cross validation fold is 1.5 times faster for ranger (378,582.6 s) than for MBMDRC (603,436.6 s), and 1.3 times faster for glmnet (465,262.4 s) than for MBMDRC.

## Discussion

In this work, we extended a known algorithm to detect interactions to a classification algorithm that has a performance comparable to two popular classifications algorithms if no interactions are present, but which clearly outperforms these if interactions are present. We have shown this by a comprehensive simulation study and by application to real data. Specifically, our simulation study revealed that our new classification algorithm can use information hidden in interactions more efficiently than the Random Forest approach, i.e. smaller sample sizes are required to achieve similar performance. The Elastic Net, at least in available implementations, does not consider interactions at all, this is inappropriate if the outcome is influenced by interacting features. In our application to the real dataset on RA, the performance of our algorithm was not relevantly different from that of the competitors, indicating that even though Liu et al. [39] claimed to have identified putative interactions on chromosome 6 and MDR models of two SNPs entered the MBMDRC models, this did not improve the classification performance. Comparing the relative performance in the real data, this result is most similar to scenario 2, i.e. five SNPs with main effects only but no interactions. However, this does not automatically mean that there are no interactions in this specific region, but first of all only that consideration of possible interactions does not improve the prediction of the disease status. That is not a contradiction, because one does not necessarily mean the other.

One drawback of our new method is the exponential increase in runtime with an increasing number of features in a dataset. Whereas its runtime is not much slower than that of the Elastic Net approach, because both depend mostly on the number of features, Random Forest is clearly the fastest one, mainly dependent on the number of samples. This makes the application of our new method to datasets on a genome-wide scale still challenging at the moment and leaves room for improvement.

As a clear strength, we have shown in our unbiased benchmark on simulated data that taking interactions into account can improve classification performance. As our method is not only applicable to biological/genetic data but to all datasets with discrete features, it may have practical implications in other applications, and we made our method available as an R package [30].

In addition, our observation that the Random Forest algorithm can make use of interacting features up to a certain degree fits well with Wright et al. [19], who conclude that Random Forest is able to capture interactions but not to detect them. In this regard, our method offers a clear advantage in that is not only able to capture interactions but the MDR models as the basic building block also allow insight into the underlying structure and dependencies among the features. Thus, our proposed algorithm can be seen as a good tradeoff between powerful prediction and interpretability of the models. In this sense our algorithm can be a valuable addition to the repertoire of methods currently used in the process of precision medicine. Providing more insight into the underlying effect structure when constructing prediction models as done with our new algorithm can in turn lead to new insights into the driving structures of the diseases analyzed.

## Conclusions

We conclude that the explicit use of interactions between features can improve the prediction performance and thus should be included in further attempts to move precision medicine forward. In addition, our algorithm offers a way to understand which feature effects influence prediction.

## Methods

Throughout this work we assume that ${n}_{D}$ samples in a dataset $D$ are characterized by $q$ independent variables ${\varvec{X}}\in {\mathcal{X}}^{{n}_{D}\times }={\mathcal{X}}_{1}^{{n}_{D}}\times \dots \times {\mathcal{X}}_{q}^{{n}_{D}}$. The independent variables are of categorical type, thus ${\mathcal{X}}_{j}={\mathbb{N}}^{+}$, e.g. genotypes of single nucleotide polymorphisms (SNP). Additionally, the dependent variable $Y\in {\mathcal{Y}}^{{n}_{D}}$ denotes the true outcome of each sample in $D$. Depending on $\mathcal{Y}$, the task is to estimate $\hat{Y}$ given ${\varvec{X}}$, thus either.

1. The class outcome $\hat{Y}=k$, i.e. classification to class $k$ out of $K$ classes ($\mathcal{Y}\in {\mathbb{N}}$),
2. The class probabilities $\hat{Y}=\hat{\mathbb{P}}\left(Y=k\mid {\varvec{X}}\right)$, i.e. probability estimation for each class $k$ out of $K$ classes ($\mathcal{Y}\in {\mathbb{N}}$),
3. Or to estimate a continuous outcome $\hat{Y}$, i.e. regression of ${\varvec{X}}$ on $Y$ ($\mathcal{Y}\in {\mathbb{R}}$).

To estimate an individual outcome $\hat{y}$ we use models ${M}_{A;{\varvec{h}}}^{D}$, based on algorithm $A$ with hyperparameter settings ${\varvec{h}}$ and trained on $D$, given a realization ${\varvec{x}}$ of the independent variables: $\hat{y}={M}_{A;{\varvec{h}}}^{D}\left({\varvec{x}}\right)$.

### Model-based multifactor dimensionality reduction (MB-MDR)

The MB-MDR algorithm was first described by Calle et al. [24]. For a detailed review of the MDR algorithm and its extensions including the MB-MDR algorithm together with detailed descriptions of the algorithms we refer to Gola et al. [23]. Here, we give only a brief overview of the MB-MDR algorithm to lay out the basics of our new algorithm to enable individual trait prediction. For a graphical illustration of the MB-MDR core algorithm we refer to Additional file 21: Figure 12, adapted from Gola et al. [23].

The MB-MDR is an extension of the MDR such that the assignment of the cell labels, i.e. the combinations of feature levels, is based on an appropriate statistical test and that each possible combination of features, i.e. MDR model, is ranked by a test statistic. Suppose each sample $i$, $i=1,\dots ,n$ is characterized by $q$ discrete features ${{\varvec{x}}}_{i}=\left({x}_{1},\dots ,{x}_{q}\right)$, with each feature ${x}_{j}$, $j=1,\dots ,q$ having ${l}_{j}$ levels. We specifically assume that all possible feature levels are known, e.g., the three possible genotypes of a single SNP. The observed outcome of each sample is denoted by ${y}_{i}$ and can be of an arbitrary scale. The core algorithm of the MB-MDR consists of five steps:

1. Select $d\le q$ features ${x}_{{j}_{k}}$ with ${l}_{{j}_{k}}$ levels, where $k=1,\dots ,d$ (${j}_{k}\in \left\{1,\dots ,q\right\}$).
2. Arrange the samples based on the selected features in the $d$-dimensional space by grouping samples with the same level combinations of the $d$ features into cells ${c}_{m}$, $m=1,\dots ,\prod_{k}^{d}{l}_{{j}_{k}}$.
3. Perform appropriate hypothesis tests with corresponding test statistics ${T}_{m}$ and $p$ values ${p}_{m}$, comparing the samples in each cell ${c}_{m}$ with all other samples not in ${c}_{m}$.
4. Assign a label to each cell ${c}_{m}$ to construct the MDR model defined by the selected features based on the respective hypothesis test:
5. If less than ${n}_{min}$ samples are in ${c}_{m}$ or ${p}_{m}\ge \alpha$, ${c}_{m}$ has an ambiguous risk and is labeled as $O$.
6. If at least ${n}_{min}$ samples are in ${c}_{m}$ and ${p}_{m}<\alpha$, the value of ${T}_{m}$ determines the label of ${c}_{m}$ as high risk ($H$) or low risk ($L$).
7. Derive a test statistic for the current MDR model by selecting the maximum test statistic of two appropriate hypothesis tests:
8. Test samples in high risk cells against all other samples.
9. Test samples in low risk cells against all other samples.

This core algorithm is repeated for all $r=1,\dots ,\left(\genfrac{}{}{0pt}{}{q}{d}\right)$ possible combinations of $d$ out of $q$ features and possibly for several values of $d$, constructing MDR models ${f}_{d,r}$. Finally, the MDR models can be sorted by their respective test statistic and using a permutation-based strategy, $p$ values can be assigned to each MDR model. Several improvements and extensions of this basic algorithm allow to analyze different outcomes, such as dichotomous [25], continuous [26] and survival [27] traits, or to adjust for covariates and lower order effects of the features of an MDR model [28]. A fast C +  + implementation of the MB-MDR is available [29] and used in this work.

### Extension of MB-MDR to individual prediction

We extended the MB-MDR algorithm to not only detect interactions between features but to allow individual predictions based on the MDR models. It is important to note that each MDR model is a prediction model in itself using $d$ features and that each cell of an MDR model includes the predicted outcome for the respective feature levels combination. Thus, after the construction of the MDR models and selection of the $s$ best MDR models, the prediction for a new sample is the aggregation of the characteristics of the cells the sample falls into. As for all MDR-based algorithms, this requires that a new sample cannot contain factor levels that were not considered in model building; in the case of SNPs as independent variables, all factor levels are known and considered in advance. In our framework, instead of calculating $p$ values of the MDR models, $s$ is determined by cross-validation during training. Here, $s$ is chosen, such that a loss, e.g. the mean squared error, is minimized or a performance measure, e.g. the AUC, is maximized.

Suppose a new sample ${i}^{*}$ with features ${{\varvec{x}}}_{{i}^{*}}$. Then, ${i}^{*}$ is a member of one specific cell ${c}_{m}$ in each of the best $s$ MDR models ${f}_{d,r}$, $r=1,\dots ,s$. Different types of predictions are possible using different cell values and aggregations.

1. Predicting a binary outcome, i.e., the classification task.
  - *Hard classification* Count the number of MDR models in which ${i}^{*}$ is a member of cells labelled as $H$ and cells labelled as $L$. Then, the estimated class of ${i}^{*}$ is the most frequent cell label among the $s$ best MDR models.
  - *Probability estimation.* The natural estimate for the probability of being member of a specific class for a new sample ${i}^{*}$, given the membership in a certain cell ${c}_{m}$ of a MDR model ${f}_{d,r}$, is the proportion of the specific class in that cell, regardless of whether if it is labelled as either $H$ or $L$. The simple average across the $s$ MDR models with the highest test statistics results in an aggregated estimate of the probability of being a case. Here, $O$ labelled cells may be treated in either of two ways:
    1. $O$ labelled cells are considered as missing values and thus are not considered in the aggregated estimate.
    2. $O$ labelled cells are included as the global estimate of the class probabilities in the training dataset.
2. *Predicting a continuous outcome, i.e., the regression task.* The same principle as in probability estimation applies to prediction in regression tasks for a continuous outcome. Here, the predicted outcome is given by the average of the mean outcome of training samples in the respective cells of the $s$ highest ranked MDR models. Again, $O$ labelled cells may be treated in either of two ways:
3. $O$ labelled cells are considered as missing values and thus are not considered in the aggregated estimate.
4. $O$ labelled cells are included as the global estimate of the mean outcome in the training dataset. Hard classification can be done by taking the most frequent cell label $H$ or $L$ among the $s$ MDR models.
5. *General risk prediction.* Additionally, a score can be constructed by counting $H$ cells as $+1$, $L$ cells as $-1$ and $O$ cells as 0. The higher the score of ${i}^{*}$, the higher the risk of the specific outcome.

The MB-MDR classification algorithm (MBMDRC) described so far has been implemented for classification tasks as function MBMDRC in the R package MBMDRClassifieR available on GitHub [30].

### Simulation study

A simulation study was performed to compare our proposed algorithm with two state-of-the-art prediction algorithms, the Random Forest [17] and the Elastic Net [31], a generalization of the LASSO [16] and ridge regression [15], for classification tasks. As implementations we utilized the R (version 3.3.1) [32] packages ranger (version 0.8.1.300) [33] and glmnet (version 2.0-5) [20]. We considered eight scenarios to investigate the performance of the three algorithms given different underlying effect structures. The scenarios start with very simple effect structures and gradually become more complex. As an example, the data generation procedure is illustrated additionally for scenario 4 in Additional file 22. In each scenario different simulation parameter combinations were considered. For each scenario and combination of simulation parameters 50 datasets $D$ were created as replicates. In each replication we independently simulated $q=100$ SNPs in total, and of those, different numbers of SNPs or combinations of SNPs are used as effect feature components:

1. One single SNP
2. Five single SNPs without interaction
3. One interaction of two SNPs
4. One interaction of two SNPs and three single SNPs without interactions
5. Two interactions of two SNPs each
6. Three interactions of two SNPs each
7. Three interactions of two SNPS each and three single SNPs without interactions
8. One interaction of three SNPs and three single SNPs without interactions

The effect strength of each component was defined by the heritability ${h}^{2}\in \left\{\mathrm{0.05,0.1,0.2}\right\}$. The minor allele frequencies (MAF) of the effect SNPs was set to 0.1, 0.2 or 0.4. The MAF of the additional SNPs were randomly selected from $\left(\mathrm{0.05,0.5}\right)$. All genotypes were simulated under the assumption of Hardy–Weinberg equilibrium. To translate the given heritability and MAF into penetrances, we generated penetrance tables of interacting SNPs, i.e. the probability of having a phenotype given a certain combination of genotypes, by the GAMETES software (version 2.1) [34] without any marginal effects of the interacting SNPs. It was not possible to generate penetrance tables for ${h}^{2}=0.2$ in scenario 8 with GAMETES, thus this setting is left out in the following. The penetrance tables of single effect SNPs were created under the restriction of rendering $\beta$ coefficients in a logistic regression model with an additive coding of the SNPs. Detailed information on the statistical background and the connection between regression model coefficients and penetrance tables can be found as additional file (see Additional file 13). In scenarios with multiple SNP combinations, the single penetrances were aggregated on the *logit* scale and transformed back to probabilities using the *expit* transformation. Phenotype, e.g. disease status, of a sample was then determined by drawing from a Bernoulli distribution with the aggregated penetrance as phenotype probability. We considered sample sizes of 200, 1000, 2000 and 10,000 with equal numbers of cases and controls.

For the benchmarking regarding the AUC of the three algorithms, we used the mlr framework (version 2.12) [35]. Each dataset $D$ was split into datasets ${D}_{1}$ and ${D}_{2}$ of the same size. Tuning was performed with fivefold cross-validation on ${D}_{1}$ using the R package mlrMBO (version 1.1.0) [36] for 100 iterations with ranger (ntrees: 500, mtry: square root of the number of tuning hyperparameters) as the surrogate learner. The hyperparameter spaces considered for tuning are shown in Table 1 together with their respective descriptions. After tuning, a prediction model with the tuned parameters was built on ${D}_{1}$ and the prediction performance was calculated on ${D}_{2}$ for each replicate.

**Table 1:** Hyperparameter spaces used for tuning

Algorithm	Hyperparameter	Description	Values
glmnet	alpha	Elastic net mixing parameter. alpha = 1 is the LASSO, alpha = 0 is the ridge penalty	$\left\{\mathrm{0,0.25,0.5,0.75,1}\right\}$
ranger	num.trees	Number of trees	1000
	mtry	Number of variables to possibly split at in each node	$\left[\mathrm{1,100}\right]\subset {\mathbb{N}}$
	min.node.size	Minimal node size	$\left[\mathrm{10,100}\right]\subset {\mathbb{N}}$
MBMDRC	min.cell.size	Minimum number of samples with a specific genotype combination to be statistically relevant. If less, a cell is automatically labelled as $O$	$\left[\mathrm{0,50}\right]\subset {\mathbb{N}}$
	alpha	Significance level used to determine $H$, $L$ and $O$ label of a cell	$\left(\mathrm{0.01,1}\right)\subset {\mathbb{R}}$
	adjustment	Adjustment for lower order marginal effects	{NONE, CODOMINANT}
	order	Number of SNPs to be considered in MDR models	$\left\{\mathrm{1,2}\right\}$
	order.range	Use order as upper limit?	{TRUE, FALSE}
	o.as.na	Use $O$ labelled cells as NA or as the global probability/mean estimate	{TRUE, FALSE}

The underlying code is available from the authors upon request.

### Application to real data

We also compared the performance of the three algorithms on a dataset by the North American Rheumatoid Arthritis Consortium (NARAC) comprised of 1194 cases with rheumatoid arthritis and 868 controls, genotyped at 545,080 SNPs, which is described in detail by Amos et al. [37]. Previously, Liu et al. [39] identified some putatively interacting loci in the HLA region on chromosome 6 in this dataset. We removed SNPs and samples with high missing rates ($>0.02$ and $>0.1$ respectively) and selected all SNPs with MAF $>0.1$ on chromosome 6 after LD pruning (window size: ${10}^{6}$ SNPs, step size: 1 SNP, ${r}^{2}$ threshold: 0.75). This resulted in a dataset of 868 cases and 1194 controls with 18,263 SNPs. As in the benchmarking on the simulated datasets, we used mlr and mlrMBO with the same settings as before in nested cross-validation with tenfold outer cross-validation.

The underlying R code is available from the authors upon request.

## Supplementary Information

**Additional file 1: Figure 1**. Performance in simulation scenario 4. Performance of the algorithms MBMDRC, RANGER, and GLMNET measured as AUC over 50 replicates in sample sizes 200, 1000, 2000, and 10,000 in scenario 4: one pair of interacting SNPs without marginal effects and three SNPs with main effects (MAF 0.1, 0.2, or 0.4 and heritability 0.05, 0.1, 0.2), 95 SNPs without any effect.

**Additional file 2: Figure 2**. Performance in simulation scenario 5. Performance of the algorithms MBMDRC, RANGER, and GLMNET measured as AUC over 50 replicates in sample sizes 200, 1000, 2000, and 10,000 in scenario 5: two pairs of interacting SNPs without marginal effects (MAF 0.1, 0.2, or 0.4 and heritability 0.05, 0.1, 0.2), 96 SNPs without any effect.

**Additional file 3: Figure 3**. Performance in simulation scenario 6. Performance of the algorithms MBMDRC, RANGER, and GLMNET measured as AUC over 50 replicates in sample sizes 200, 1000, 2000, and 10,000 in scenario 6: three pairs of interacting SNPs without marginal effects (MAF 0.1, 0.2, or 0.4 and heritability 0.05, 0.1, 0.2), 94 SNPs without any effect.

**Additional file 4:** **Table 1**. Performance in scenario 1. Performance of the algorithms MBMDRC, RANGER, and GLMNET measured as AUC over 50 replicates in scenario 1: one SNP with main effect (MAF 0.1, 0.2, or 0.4 and heritability 0.05, 0.1, 0.2), 99 SNPs without any effect.. The median of the AUC and the 25% and 75% quantile in parentheses over 50 replicates are given.

**Additional file 5: Table 2**. Performance in scenario 2. Performance of the algorithms MBMDRC, RANGER, and GLMNET measured as AUC over 50 replicates in scenario 2: five SNPs with main effects (MAF 0.1, 0.2, or 0.4 and heritability 0.05, 0.1, 0.2), 95 SNPs without any effect.. The median of the AUC and the 25% and 75% quantile in parentheses over 50 replicates are given.

**Additional file 6: Table 3**. Performance in scenario 3. Performance of the algorithms MBMDRC, RANGER, and GLMNET measured as AUC over 50 replicates in scenario 3: one pair of interacting SNPs without marginal effects (MAF 0.1, 0.2, or 0.4 and heritability 0.05, 0.1, 0.2), 98 SNPs without any effect. The median of the AUC and the 25% and 75% quantile in parentheses over 50 replicates are given.

**Additional file 7: Table 4**. Performance in scenario 4. Performance of the algorithms MBMDRC, RANGER, and GLMNET measured as AUC over 50 replicates in scenario 4: one pair of interacting SNPs without marginal effects and three SNPs with main effects (MAF 0.1, 0.2, or 0.4 and heritability 0.05, 0.1, 0.2), 95 SNPs without any effect.. The median of the AUC and the 25% and 75% quantile in parentheses over 50 replicates are given.

**Additional file 8: Table 5**. Performance in scenario 5. Performance of the algorithms MBMDRC, RANGER, and GLMNET measured as AUC over 50 replicates in scenario 5: two pairs of interacting SNPs without marginal effects (MAF 0.1, 0.2, or 0.4 and heritability 0.05, 0.1, 0.2), 96 SNPs without any effect.. The median of the AUC and the 25% and 75% quantile in parentheses over 50 replicates are given.

**Additional file 9: Table 6**. Performance in scenario 6. Performance of the algorithms MBMDRC, RANGER, and GLMNET measured as AUC over 50 replicates in scenario 6: three pairs of interacting SNPs without marginal effects (MAF 0.1, 0.2, or 0.4 and heritability 0.05, 0.1, 0.2), 94 SNPs without any effect.. The median of the AUC and the 25% and 75% quantile in parentheses over 50 replicates are given.

**Additional file 10: Table 7**. Performance in scenario 7. Performance of the algorithms MBMDRC, RANGER, and GLMNET measured as AUC over 50 replicates in scenario 7: three pairs of interacting SNPs without marginal effects and three SNPs with marginal effects only (MAF 0.1, 0.2, or 0.4 and heritability 0.05, 0.1, 0.2), 91 SNPs without any effect.. The median of the AUC and the 25% and 75% quantile in parentheses over 50 replicates are given.

**Additional file 11: Table 8**. Performance in scenario 8. Performance of the algorithms MBMDRC, RANGER, and GLMNET measured as AUC over 50 replicates in scenario 8: three interacting SNPs without marginal effects and three SNPs with marginal effects only (MAF 0.1, 0.2, or 0.4 and heritability 0.05, 0.1, 0.2), 94 SNPs without any effect.. The median of the AUC and the 25% and 75% quantile in parentheses over 50 replicates are given.

**Additional file 12:** Statistical background. Detailed information on the statistical background and the connection between regression model coefficients and penetrance tables.

**Additional file 13: Figure 4**. ROC curves in scenario 1. ROC curves of the algorithms MBMDRC, RANGER, and GLMNET for 10,000 samples in scenario 1: one SNP with main effect (MAF 0.1, 0.2, or 0.4 and heritability 0.05, 0.1, 0.2), 99 SNPs without any effect. Light lines represent the ROC curve of each of the 50 replicates, strong lines are based on the mean true positive and true negative rates of the 50 replicates for each of a sequence of 1000 thresholds.

**Additional file 14: Figure 5**. ROC curves in scenario 2. ROC curves of the algorithms MBMDRC, RANGER, and GLMNET for 10,000 samples in scenario 2: five SNPs with main effects (MAF 0.1, 0.2, or 0.4 and heritability 0.05, 0.1, 0.2), 95 SNPs without any effect. Light lines represent the ROC curve of each of the 50 replicates, strong lines are based on the mean true positive and true negative rates of the 50 replicates for each of a sequence of 1000 thresholds.

**Additional file 15: Figure 6**. ROC curves in scenario 3. ROC curves of the algorithms MBMDRC, RANGER, and GLMNET for 10,000 samples in scenario 3: one pair of interacting SNPs without marginal effects (MAF 0.1, 0.2, or 0.4 and heritability 0.05, 0.1, 0.2), 98 SNPs without any effect. Light lines represent the ROC curve of each of the 50 replicates, strong lines are based on the mean true positive and true negative rates of the 50 replicates for each of a sequence of 1000 thresholds.

**Additional file 16: Figure 7**. ROC curves in scenario 4. Description: ROC curves of the algorithms MBMDRC, RANGER, and GLMNET for 10,000 samples in scenario 4: three pairs of interacting SNPs without marginal effects and three SNPs with marginal effects only (MAF 0.1, 0.2, or 0.4 and heritability 0.05, 0.1, 0.2), 91 SNPs without any effect. Light lines represent the ROC curve of each of the 50 replicates, strong lines are based on the mean true positive and true negative rates of the 50 replicates for each of a sequence of 1000 thresholds.

**Additional file 17: Figure 8**. ROC curves in scenario 5. ROC curves of the algorithms MBMDRC, RANGER, and GLMNET for 10,000 samples in scenario 5: two pairs of interacting SNPs without marginal effects (MAF 0.1, 0.2, or 0.4 and heritability 0.05, 0.1, 0.2), 96 SNPs without any effect. Light lines represent the ROC curve of each of the 50 replicates, strong lines are based on the mean true positive and true negative rates of the 50 replicates for each of a sequence of 1000 thresholds.

**Additional file 18: Figure 9**. ROC curves in scenario 6. ROC curves of the algorithms MBMDRC, RANGER, and GLMNET for 10,000 samples in scenario 6: three pairs of interacting SNPs without marginal effects (MAF 0.1, 0.2, or 0.4 and heritability 0.05, 0.1, 0.2), 94 SNPs without any effect. Light lines represent the ROC curve of each of the 50 replicates, strong lines are based on the mean true positive and true negative rates of the 50 replicates for each of a sequence of 1000 thresholds.

**Additional file 19: Figure 10**. ROC curves in scenario 7. ROC curves of the algorithms MBMDRC, RANGER, and GLMNET for 10,000 samples in scenario 7: three pairs of interacting SNPs without marginal effects and three SNPs with marginal effects only (MAF 0.1, 0.2, or 0.4 and heritability 0.05, 0.1, 0.2), 91 SNPs without any effect. Light lines represent the ROC curve of each of the 50 replicates, strong lines are based on the mean true positive and true negative rates of the 50 replicates for each of a sequence of 1000 thresholds.

**Additional file 20: Figure 11**. ROC curves in scenario 8. ROC curves of the algorithms MBMDRC, RANGER, and GLMNET for 10,000 samples in scenario 8: three interacting SNPs without marginal effects and three SNPs with marginal effects only (MAF 0.1, 0.2, or 0.4 and heritability 0.05, 0.1, 0.2), 94 SNPs without any effect. Light lines represent the ROC curve of each of the 50 replicates, strong lines are based on the mean true positive and true negative rates of the 50 replicates for each of a sequence of 1000 thresholds.

**Additional file 21: Figure 12**. Illustration of MB-MDR core algorithm. Step (1): $d=2$ features are selected. Step (2): All samples, in this example cases and controls, in the dataset are arranged based on the selected features in the $d$-dimensional space by grouping samples with the same level combinations of the $d$ features into cells ${c}_{1},\dots ,{c}_{9}$. Step (3): Calculation of ${\chi }^{2}$-test statistics in each of the cells by comparing the cases and controls in the cell with all other samples not in the cell. Step (4): Assign an $O$ label to a cell if the respective ${\chi }^{2}$-test statistic from the previous step is less than ${\chi }_{1}^{2}\left(1-\alpha \right)$, otherwise a high risk ($H$, more cases than controls in the cell) or low risk ($L$, less cases than controls in the cell) label. Step (5): Derive a test statistic for the current MDR model by selecting the maximum test statistic of two ${\chi }^{2}$-tests: 1. comparing samples in high risk cells against all other samples, 2. Comparing samples in low risk cells against all other samples. Figure adapted from Gola et al. [23].

**Additional file 22:** Illustration of data generation procedure. In scenario 4 one interaction of two SNPs and three single SNPs build up the underlying effect structure. In each replicate SNP data of an unlimited population is generated according to the MAF specifications, here 0.2 for *L*_*1*_ and *L*_*2*_, 0.1 for *L*_*3*_, 0.2 for *L*_*4*_, and 0.4 for *L*_*5*_. Penetrance tables are generated according to the scenario MAF and heritability specifications, i.e. *h*^*2*^ = 0.2. At each locus the penetrances according to the genotypes are added on the logit scale and transformed back to the probability scale using the expit function to create the total probability *p*_Total_. The phenotype (case or control) is sampled from a Bernoulli distribution with success probability *p*_Total_. From the population a random sample of cases and controls is drawn from the replication dataset *D*.

## Data Availability

All data generated during this study are included in this published article and its supplementary information files. The dataset on rheumatoid arthritis used during the current study is owned by the North American Rheumatoid Arthritis Consortium and is available from the corresponding author of [37] on reasonable request.

## Abbreviations

AUC: Area under the receiver operating characteristic curve
MAF: Minor allele frequency
MB-MDR: Model-based multifactor dimensionality reduction
MBMDRC: Model-based multifactor dimensionality reduction classification algorithm
MDR: Multifactor dimensionality reduction
NARAC: North American Rheumatoid Arthritis Consortium
SNP: Single nucleotide polymorphism
