# Supplementary figures and images for: Empowering individual trait prediction using interactions for precision medicine
Source: BMC Bioinformatics. 2021 Feb 18;22:74. doi: 10.1186/s12859-021-04011-z (PMC7890638; doi:10.1186/s12859-021-04011-z)

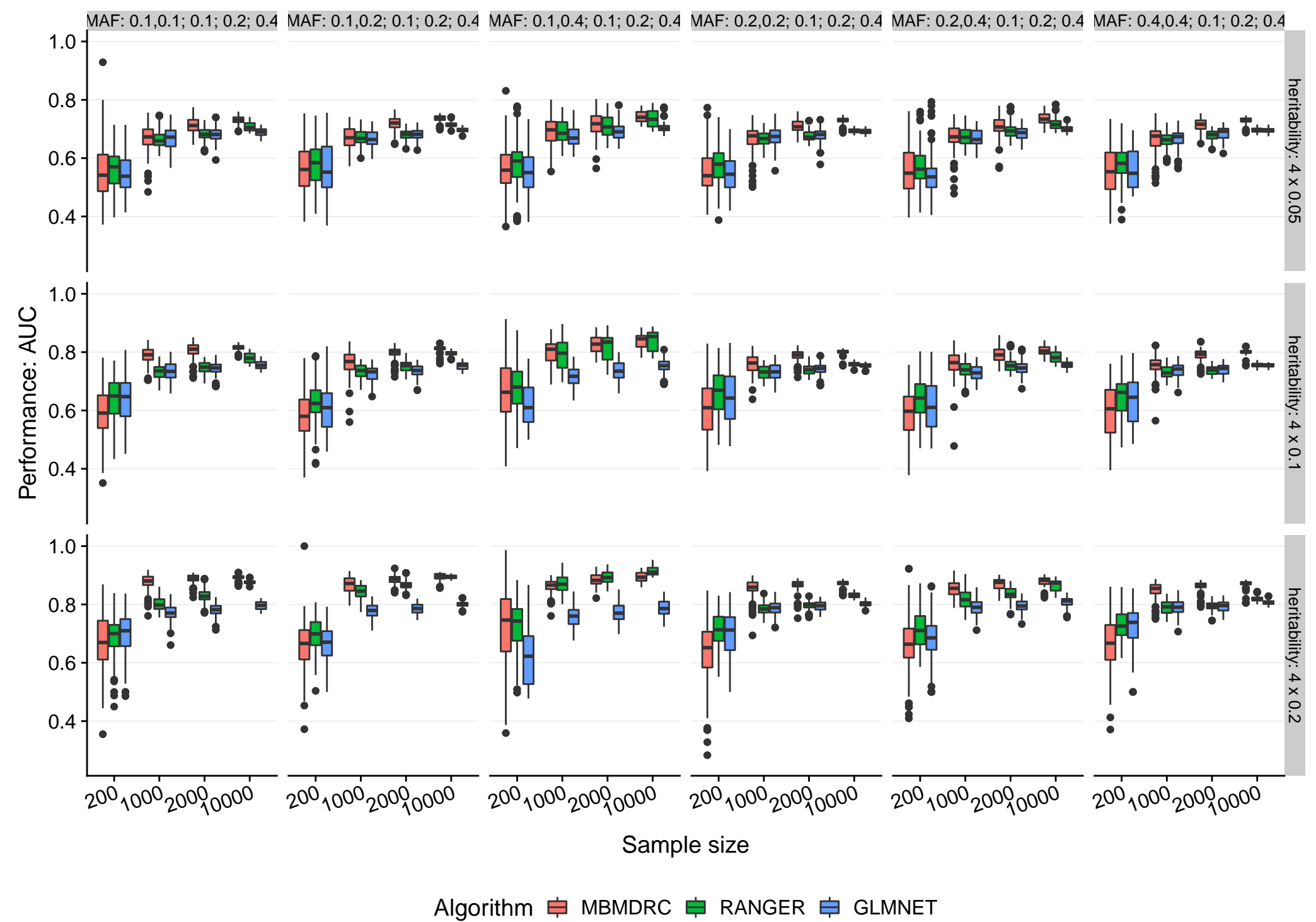

Supplement: Supplementary file 1 — Additional file 1: Figure 1. Performance in simulation scenario 4. Performance of the algorithms MBMDRC, RANGER, and GLMNET measured as AUC over 50 replicates in sample sizes 200, 1000, 2000, and 10,000 in scenario 4: one pair of interacting SNPs without marginal effects and three SNPs with main effects (MAF 0.1, 0.2, or 0.4 and heritability 0.05, 0.1, 0.2), 95 SNPs without any effect. [file 12859_2021_4011_MOESM1_ESM.pdf]

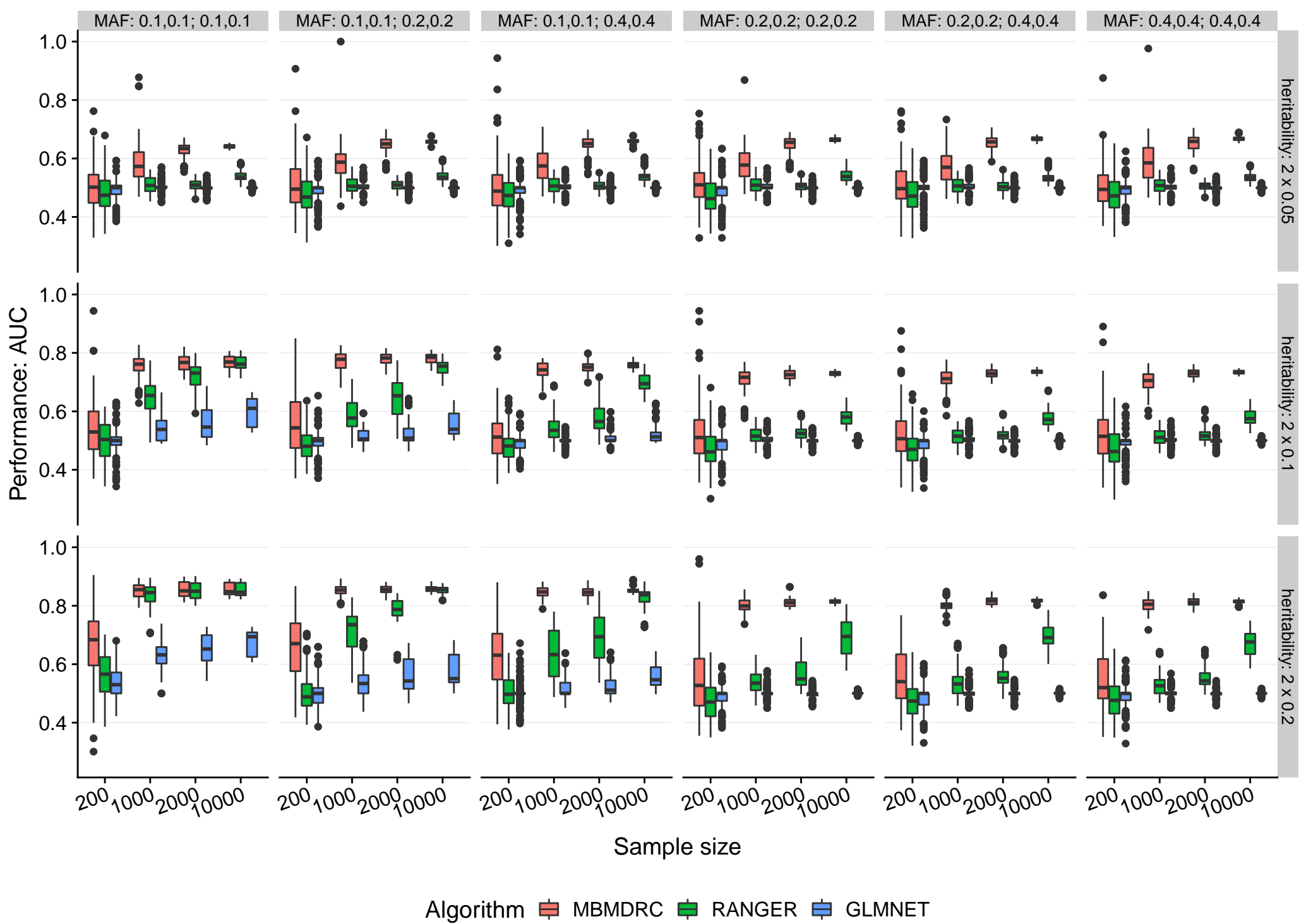

Supplement: Supplementary file 2 — Additional file 2: Figure 2. Performance in simulation scenario 5. Performance of the algorithms MBMDRC, RANGER, and GLMNET measured as AUC over 50 replicates in sample sizes 200, 1000, 2000, and 10,000 in scenario 5: two pairs of interacting SNPs without marginal effects (MAF 0.1, 0.2, or 0.4 and heritability 0.05, 0.1, 0.2), 96 SNPs without any effect. [file 12859_2021_4011_MOESM2_ESM.pdf]

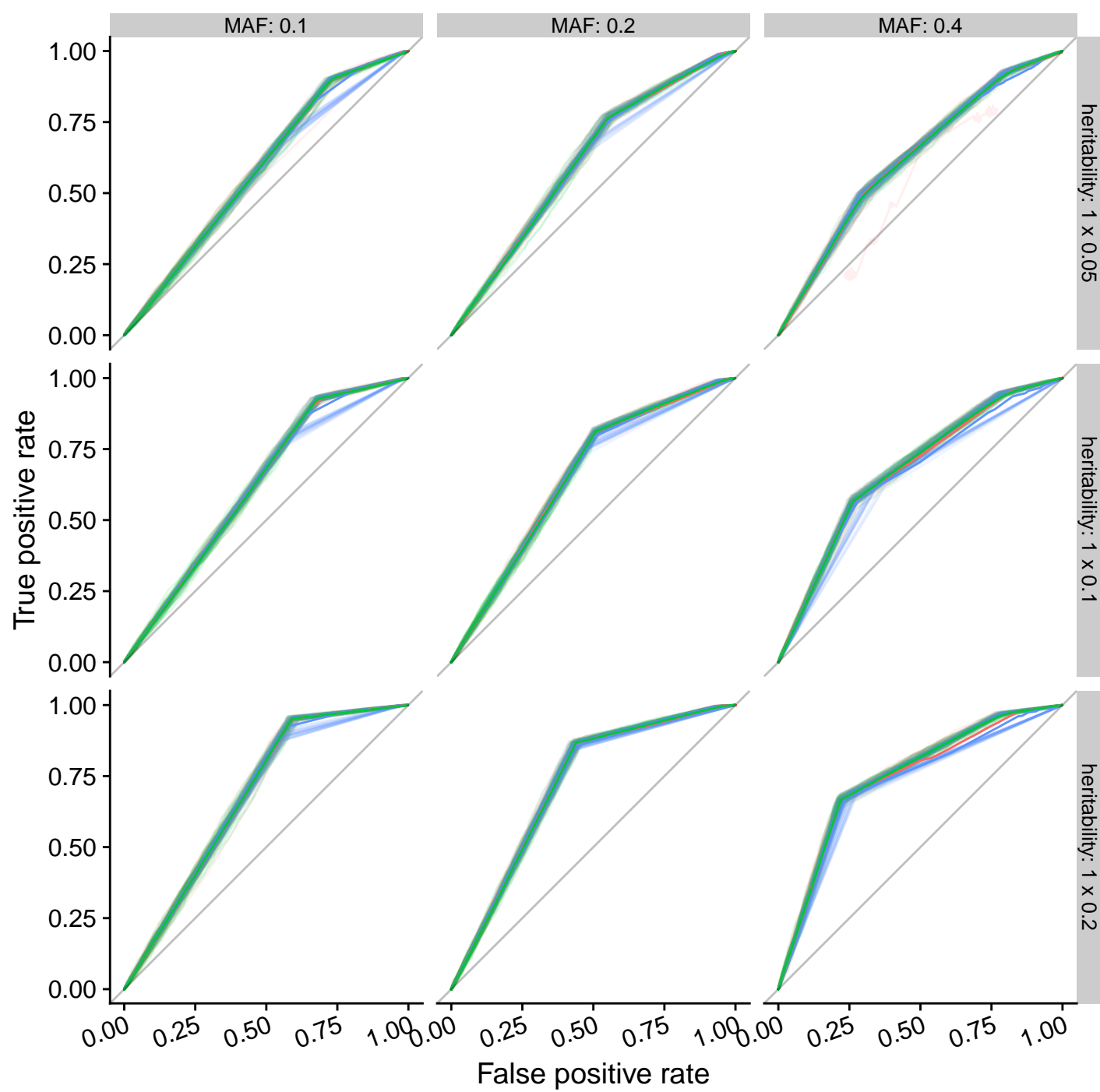

Algorithm — MBMDRC — RANGER — GLMNET

Supplement: Supplementary file 13 — Additional file 13: Figure 4. ROC curves in scenario 1. ROC curves of the algorithms MBMDRC, RANGER, and GLMNET for 10,000 samples in scenario 1: one SNP with main effect (MAF 0.1, 0.2, or 0.4 and heritability 0.05, 0.1, 0.2), 99 SNPs without any effect. Light lines represent the ROC curve of each of the 50 replicates, strong lines are based on the mean true positive and true negative rates of the 50 replicates for each of a sequence of 1000 thresholds. [file 12859_2021_4011_MOESM13_ESM.pdf]

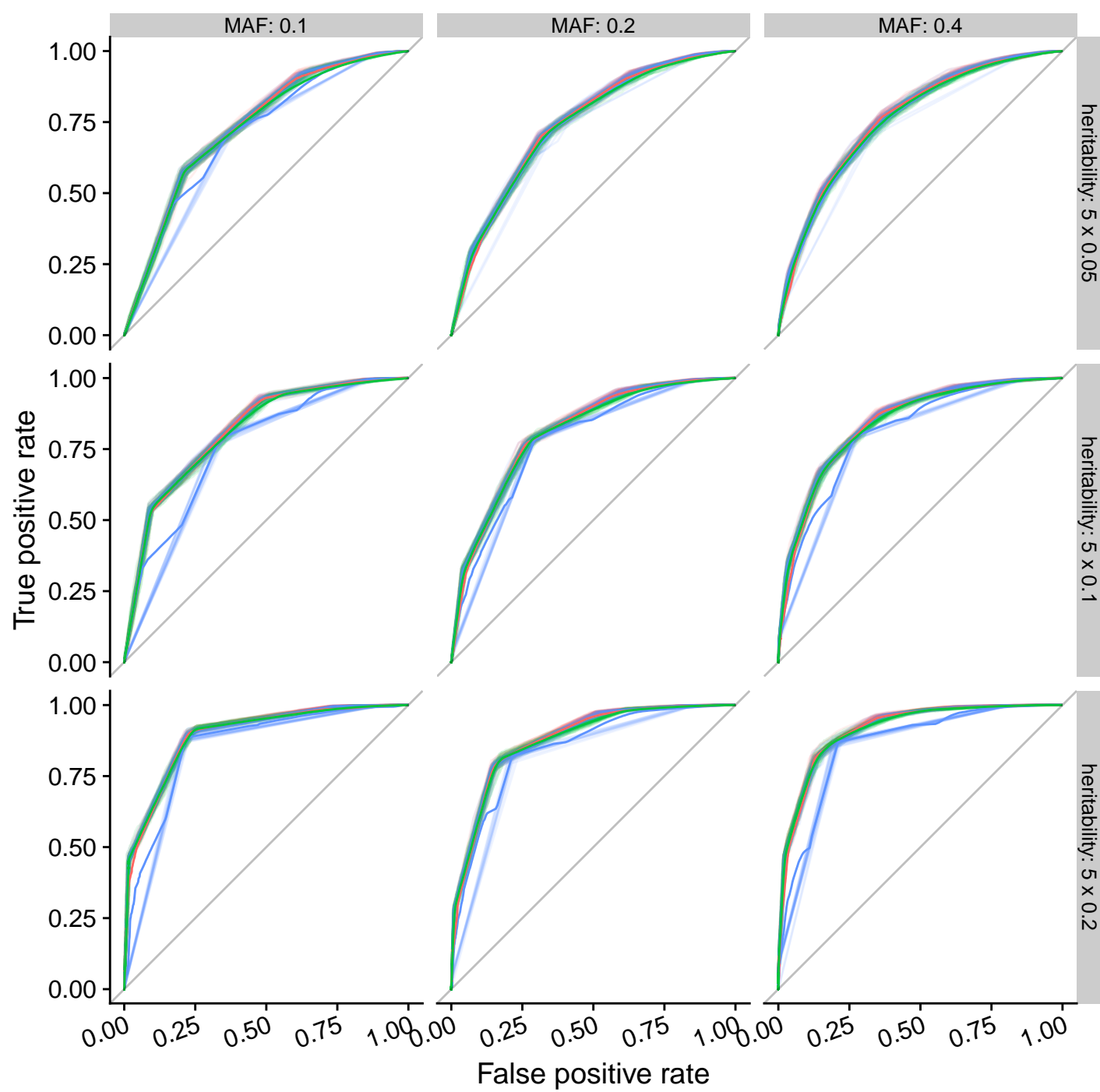

Algorithm — MBMDRC — RANGER — GLMNET

Supplement: Supplementary file 14 — Additional file 14: Figure 5. ROC curves in scenario 2. ROC curves of the algorithms MBMDRC, RANGER, and GLMNET for 10,000 samples in scenario 2: five SNPs with main effects (MAF 0.1, 0.2, or 0.4 and heritability 0.05, 0.1, 0.2), 95 SNPs without any effect. Light lines represent the ROC curve of each of the 50 replicates, strong lines are based on the mean true positive and true negative rates of the 50 replicates for each of a sequence of 1000 thresholds. [file 12859_2021_4011_MOESM14_ESM.pdf]

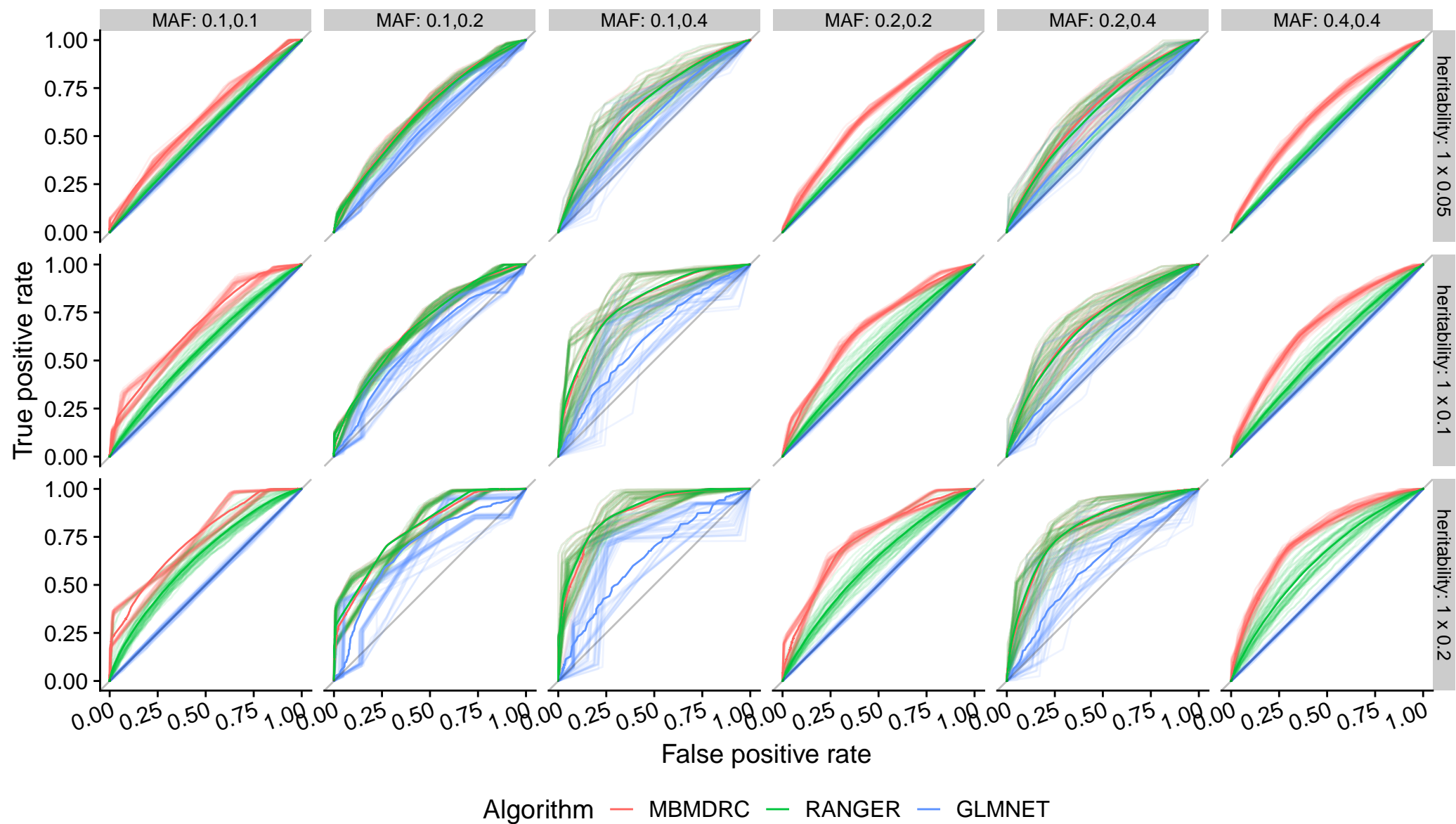

Supplement: Supplementary file 15 — Additional file 15: Figure 6. ROC curves in scenario 3. ROC curves of the algorithms MBMDRC, RANGER, and GLMNET for 10,000 samples in scenario 3: one pair of interacting SNPs without marginal effects (MAF 0.1, 0.2, or 0.4 and heritability 0.05, 0.1, 0.2), 98 SNPs without any effect. Light lines represent the ROC curve of each of the 50 replicates, strong lines are based on the mean true positive and true negative rates of the 50 replicates for each of a sequence of 1000 thresholds. [file 12859_2021_4011_MOESM15_ESM.pdf]

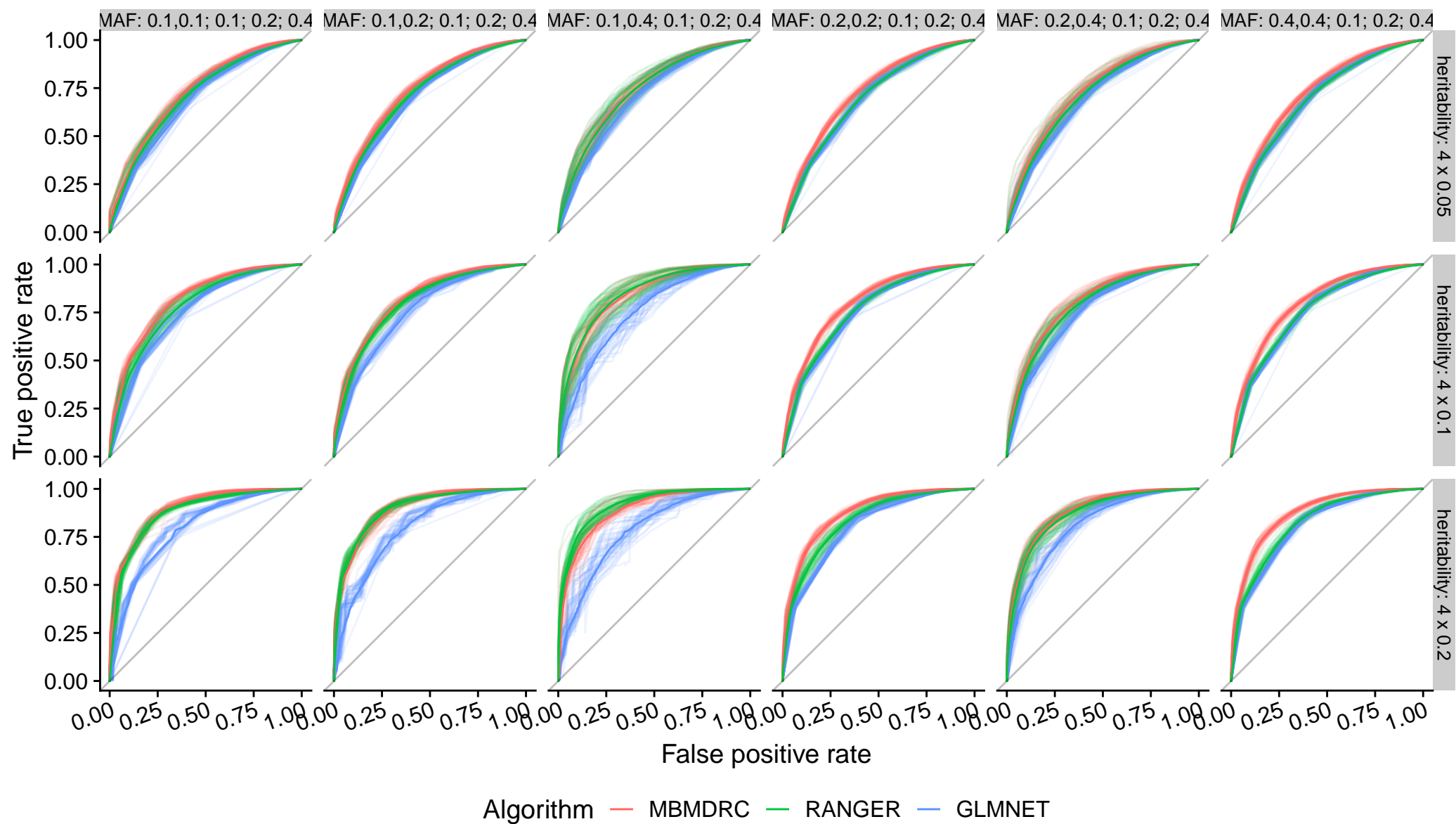

Supplement: Supplementary file 16 — Additional file 16: Figure 7. ROC curves in scenario 4. Description: ROC curves of the algorithms MBMDRC, RANGER, and GLMNET for 10,000 samples in scenario 4: three pairs of interacting SNPs without marginal effects and three SNPs with marginal effects only (MAF 0.1, 0.2, or 0.4 and heritability 0.05, 0.1, 0.2), 91 SNPs without any effect. Light lines represent the ROC curve of each of the 50 replicates, strong lines are based on the mean true positive and true negative rates of the 50 replicates for each of a sequence of 1000 thresholds. [file 12859_2021_4011_MOESM16_ESM.pdf]

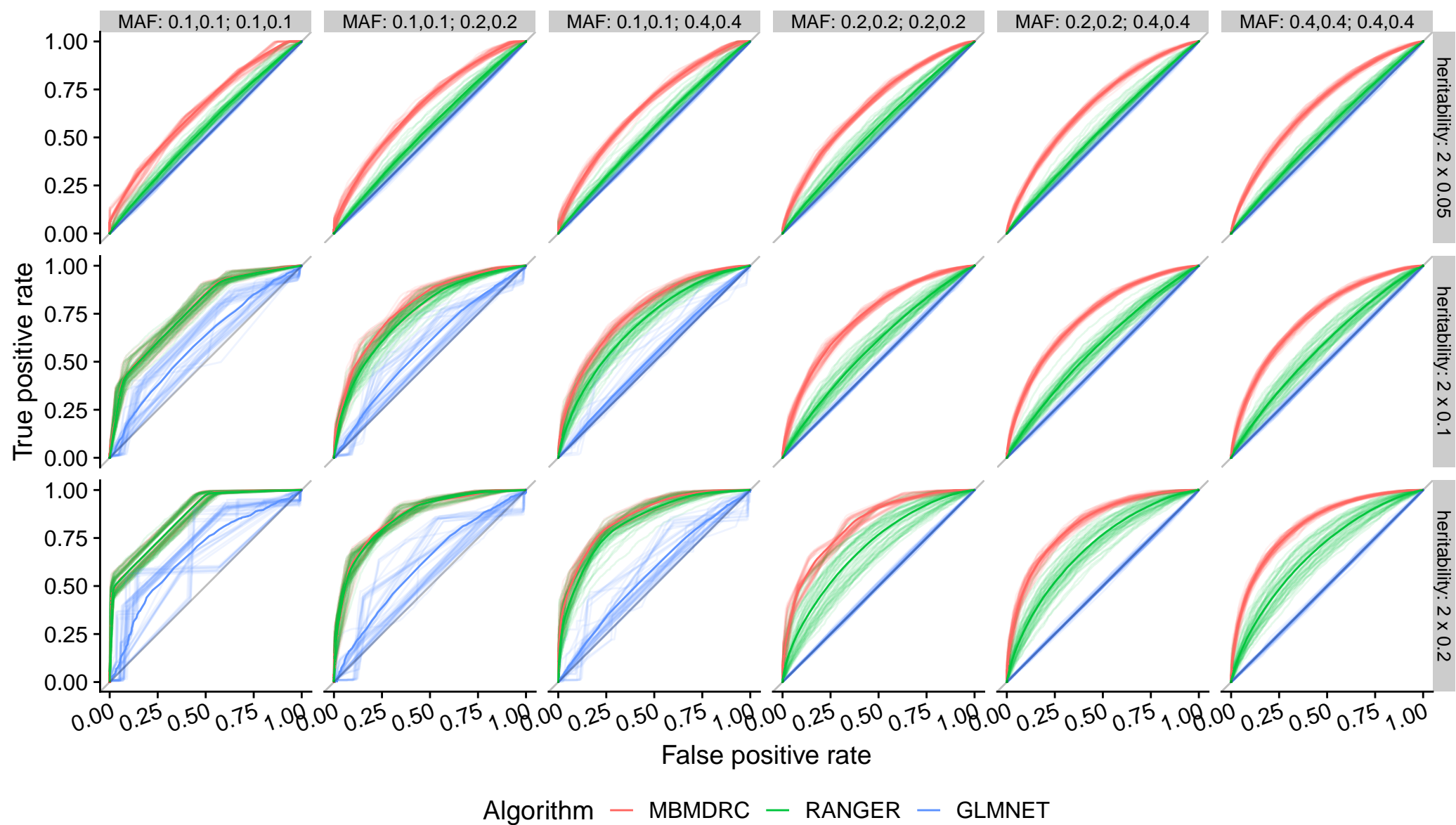

Supplement: Supplementary file 17 — Additional file 17: Figure 8. ROC curves in scenario 5. ROC curves of the algorithms MBMDRC, RANGER, and GLMNET for 10,000 samples in scenario 5: two pairs of interacting SNPs without marginal effects (MAF 0.1, 0.2, or 0.4 and heritability 0.05, 0.1, 0.2), 96 SNPs without any effect. Light lines represent the ROC curve of each of the 50 replicates, strong lines are based on the mean true positive and true negative rates of the 50 replicates for each of a sequence of 1000 thresholds. [file 12859_2021_4011_MOESM17_ESM.pdf]

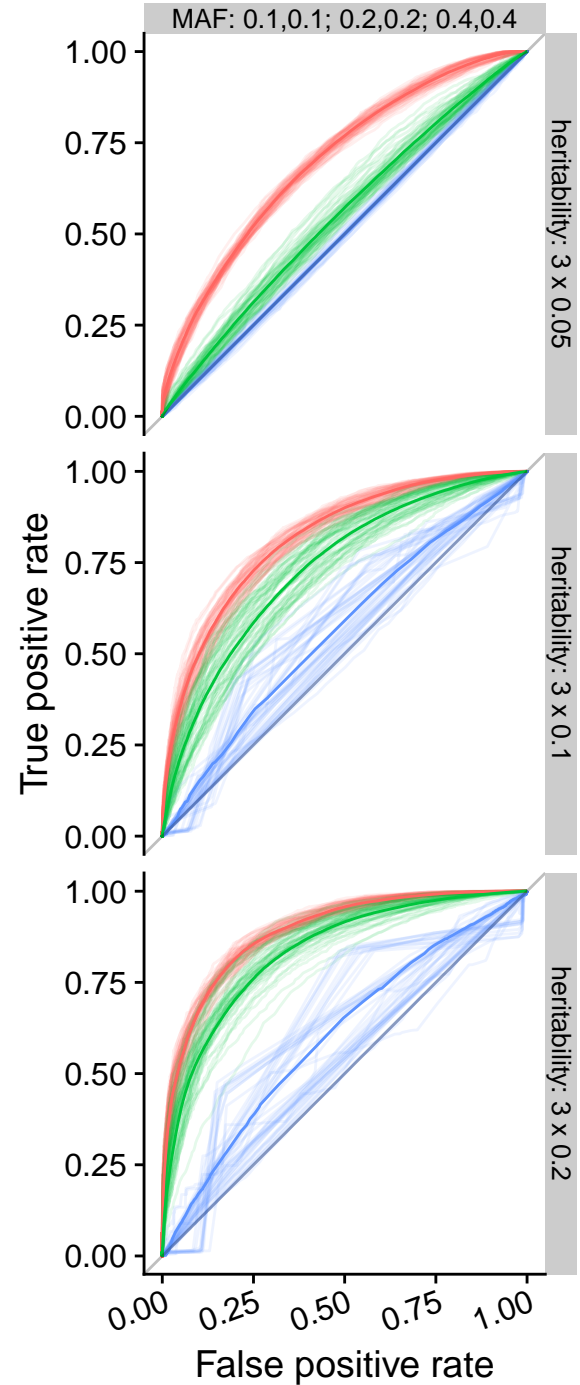

Algorithm — MBMDRC — RANGER — GLMNET

Supplement: Supplementary file 18 — Additional file 18: Figure 9. ROC curves in scenario 6. ROC curves of the algorithms MBMDRC, RANGER, and GLMNET for 10,000 samples in scenario 6: three pairs of interacting SNPs without marginal effects (MAF 0.1, 0.2, or 0.4 and heritability 0.05, 0.1, 0.2), 94 SNPs without any effect. Light lines represent the ROC curve of each of the 50 replicates, strong lines are based on the mean true positive and true negative rates of the 50 replicates for each of a sequence of 1000 thresholds. [file 12859_2021_4011_MOESM18_ESM.pdf]

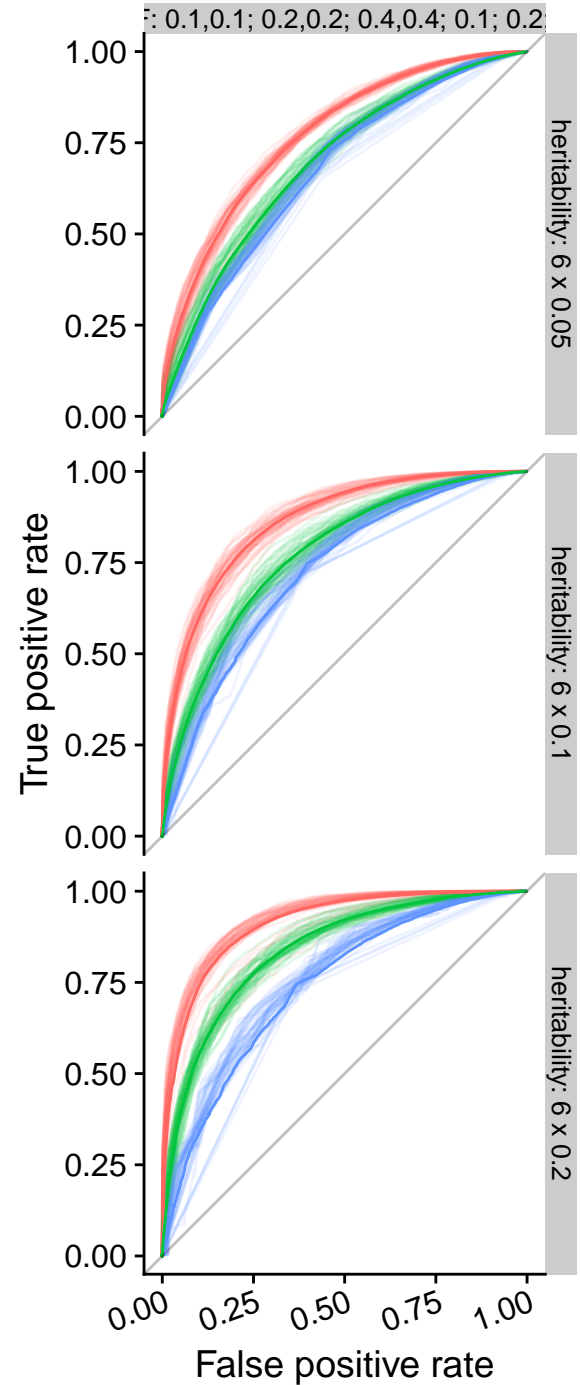

Algorithm MBMDRC RANGER GLMNET

Supplement: Supplementary file 19 — Additional file 19: Figure 10. ROC curves in scenario 7. ROC curves of the algorithms MBMDRC, RANGER, and GLMNET for 10,000 samples in scenario 7: three pairs of interacting SNPs without marginal effects and three SNPs with marginal effects only (MAF 0.1, 0.2, or 0.4 and heritability 0.05, 0.1, 0.2), 91 SNPs without any effect. Light lines represent the ROC curve of each of the 50 replicates, strong lines are based on the mean true positive and true negative rates of the 50 replicates for each of a sequence of 1000 thresholds. [file 12859_2021_4011_MOESM19_ESM.pdf]

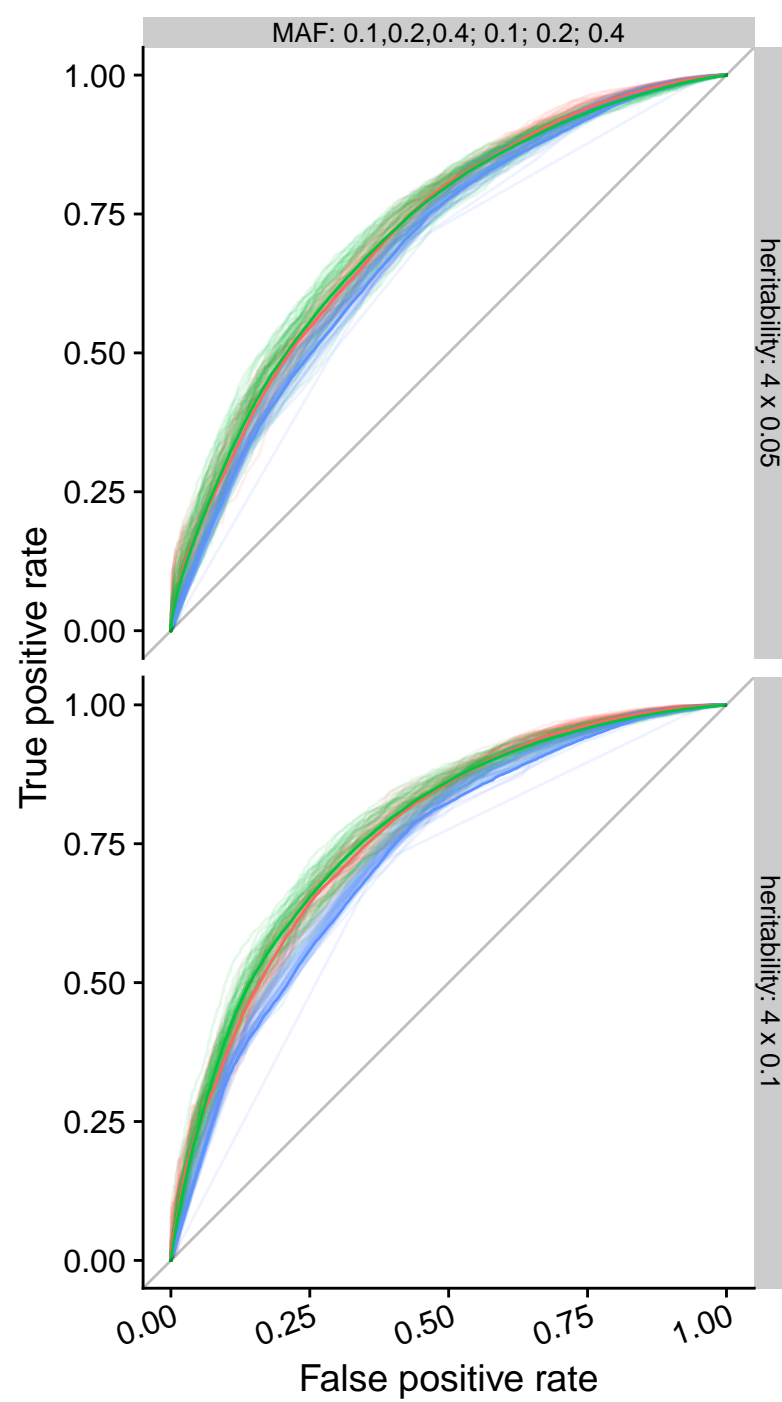

Algorithm — MBMDRC — RANGER — GLMNET

Supplement: Supplementary file 20 — Additional file 20: Figure 11. ROC curves in scenario 8. ROC curves of the algorithms MBMDRC, RANGER, and GLMNET for 10,000 samples in scenario 8: three interacting SNPs without marginal effects and three SNPs with marginal effects only (MAF 0.1, 0.2, or 0.4 and heritability 0.05, 0.1, 0.2), 94 SNPs without any effect. Light lines represent the ROC curve of each of the 50 replicates, strong lines are based on the mean true positive and true negative rates of the 50 replicates for each of a sequence of 1000 thresholds. [file 12859_2021_4011_MOESM20_ESM.pdf]

$$d = \boxed{2}, 3, \dots$$

Step (1)

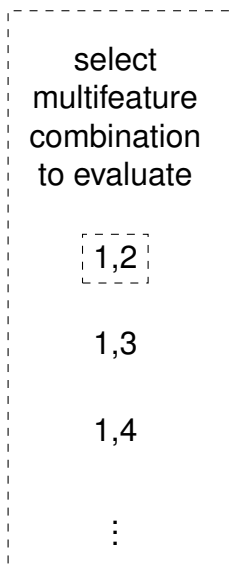

Step (2)

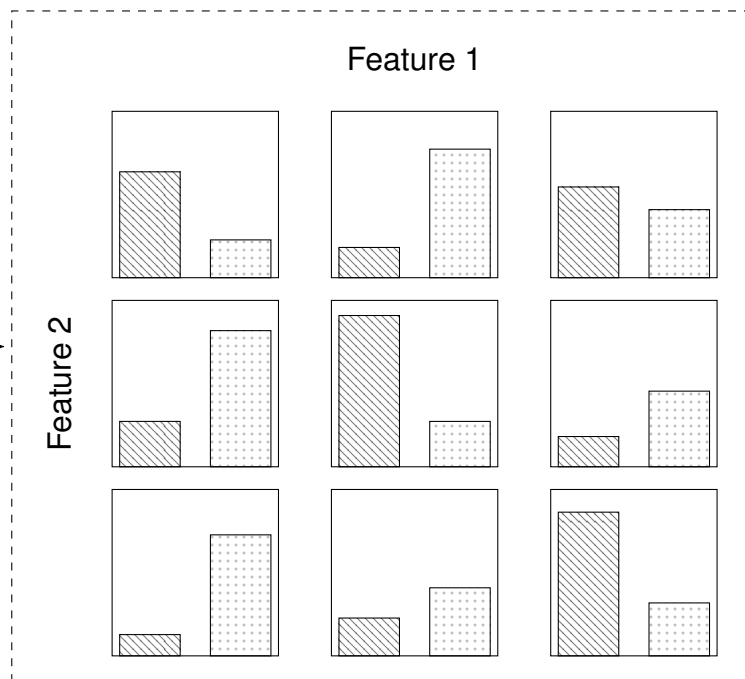

Step (3)

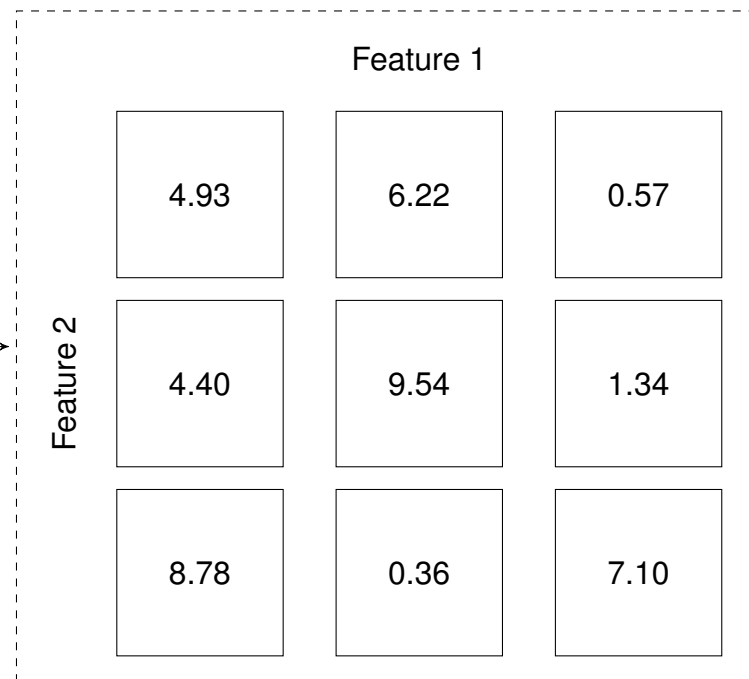

Step (4)

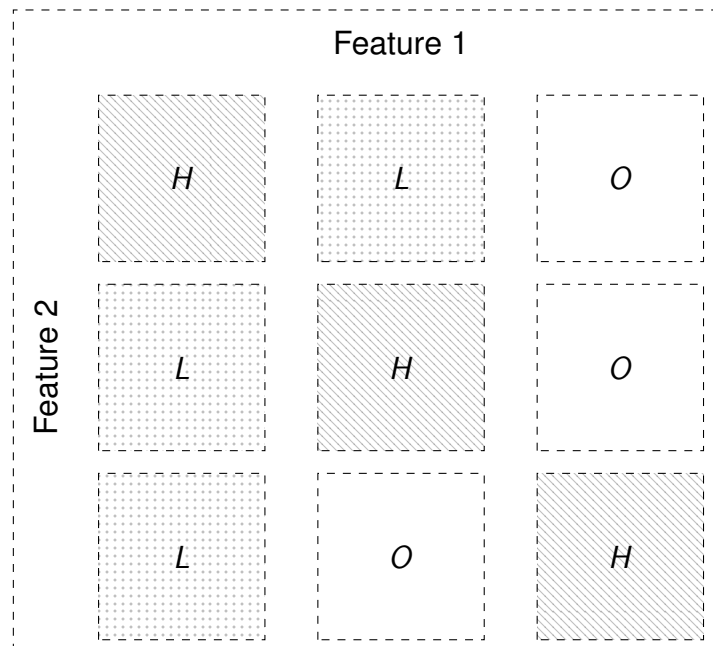

$$T > \chi_1^2(1 - \alpha)$$

Supplement: Supplementary file 21 — Additional file 21: Figure 12. Illustration of MB-MDR core algorithm. Step (1): $d=2$d=2 features are selected. Step (2): All samples, in this example cases and controls, in the dataset are arranged based on the selected features in the $d$d-dimensional space by grouping samples with the same level combinations of the $d$d features into cells ${c}_{1},\dots ,{c}_{9}$c1,⋯,c9. Step (3): Calculation of ${\chi }^{2}$χ2-test statistics in each of the cells by comparing the cases and controls in the cell with all other samples not in the cell. Step (4): Assign an [file 12859_2021_4011_MOESM21_ESM.pdf]
