# Supplementary material for: Empowering individual trait prediction using interactions for precision medicine
Source: BMC Bioinformatics. 2021 Feb 18;22:74. doi: 10.1186/s12859-021-04011-z (PMC7890638; doi:10.1186/s12859-021-04011-z)
Supplement: Supplementary file 3 — Additional file 3: Figure 3. Performance in simulation scenario 6. Performance of the algorithms MBMDRC, RANGER, and GLMNET measured as AUC over 50 replicates in sample sizes 200, 1000, 2000, and 10,000 in scenario 6: three pairs of interacting SNPs without marginal effects (MAF 0.1, 0.2, or 0.4 and heritability 0.05, 0.1, 0.2), 94 SNPs without any effect. [file 12859_2021_4011_MOESM3_ESM.pdf]

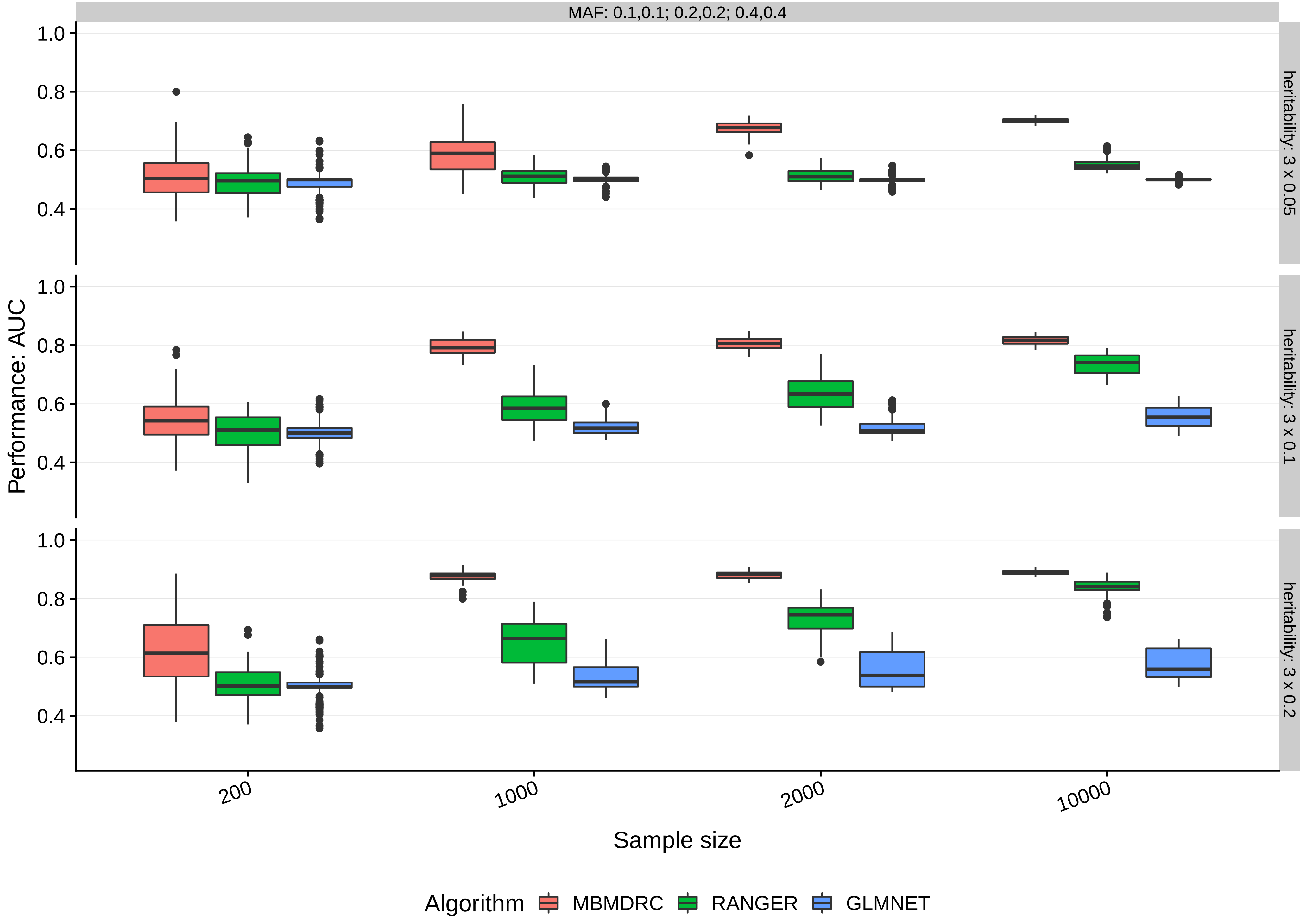

MAF: 0.1,0.1; 0.2,0.2; 0.4,0.4
Performance: AUC
1.0
0.8
0.6
0.4
heritability: 3 x 0.05
heritability: 3 x 0.1
heritability: 3 x 0.2
200
1000
2000
10000
Sample size
Algorithm
MBMDRC
RANGER
GLMNET
