# Supplementary material for: Empowering individual trait prediction using interactions for precision medicine
Source: BMC Bioinformatics. 2021 Feb 18;22:74. doi: 10.1186/s12859-021-04011-z (PMC7890638; doi:10.1186/s12859-021-04011-z)
Supplement: Supplementary file 4 — Additional file 4: Table 1. Performance in scenario 1. Performance of the algorithms MBMDRC, RANGER, and GLMNET measured as AUC over 50 replicates in scenario 1: one SNP with main effect (MAF 0.1, 0.2, or 0.4 and heritability 0.05, 0.1, 0.2), 99 SNPs without any effect.. The median of the AUC and the 25% and 75% quantile in parentheses over 50 replicates are given. [file 12859_2021_4011_MOESM4_ESM.pdf]

Table 8 Performance in scenario 1.

MAF	h^2	n	MBMDRC	RANGER	GLMNET
0.1	1 x 0.05	200	0.5391 (0.4940; 0.5858)	0.5390 (0.5001; 0.5773)	0.5316 (0.5000; 0.5854)
0.1	1 x 0.05	1000	0.5763 (0.5508; 0.5905)	0.5855 (0.5716; 0.5952)	0.5852 (0.5727; 0.5936)
0.1	1 x 0.05	2000	0.5825 (0.5678; 0.5934)	0.5841 (0.5747; 0.6009)	0.5880 (0.5781; 0.5934)
0.1	1 x 0.05	10000	0.5881 (0.5831; 0.5926)	0.5874 (0.5813; 0.5923)	0.5883 (0.5836; 0.5912)
0.1	1 x 0.1	200	0.5863 (0.5300; 0.6391)	0.6142 (0.5665; 0.6423)	0.6000 (0.5000; 0.6314)
0.1	1 x 0.1	1000	0.6117 (0.5927; 0.6355)	0.6215 (0.6049; 0.6328)	0.6129 (0.6053; 0.6297)
0.1	1 x 0.1	2000	0.6196 (0.6098; 0.6316)	0.6239 (0.6102; 0.6342)	0.6233 (0.6153; 0.6314)
0.1	1 x 0.1	10000	0.6233 (0.6206; 0.6279)	0.6239 (0.6195; 0.6279)	0.6246 (0.6207; 0.6278)
0.1	1 x 0.2	200	0.6503 (0.6041; 0.7003)	0.6800 (0.6544; 0.7046)	0.6702 (0.6408; 0.7011)
0.1	1 x 0.2	1000	0.6737 (0.6585; 0.6859)	0.6771 (0.6654; 0.6929)	0.6750 (0.6682; 0.6830)
0.1	1 x 0.2	2000	0.6765 (0.6664; 0.6875)	0.6772 (0.6686; 0.6910)	0.6801 (0.6674; 0.6908)
0.1	1 x 0.2	10000	0.6814 (0.6772; 0.6864)	0.6813 (0.6764; 0.6859)	0.6817 (0.6763; 0.6842)
0.2	1 x 0.05	200	0.5321 (0.4633; 0.5679)	0.5454 (0.4958; 0.5774)	0.5000 (0.5000; 0.5725)
0.2	1 x 0.05	1000	0.5940 (0.5697; 0.6155)	0.5981 (0.5791; 0.6148)	0.6040 (0.5904; 0.6215)
0.2	1 x 0.05	2000	0.6022 (0.5905; 0.6197)	0.6080 (0.5946; 0.6180)	0.6104 (0.6013; 0.6220)
0.2	1 x 0.05	10000	0.6103 (0.6063; 0.6138)	0.6099 (0.6068; 0.6137)	0.6095 (0.6058; 0.6130)
0.2	1 x 0.1	200	0.6021 (0.5410; 0.6523)	0.6216 (0.5665; 0.6706)	0.6144 (0.5000; 0.6646)
0.2	1 x 0.1	1000	0.6529 (0.6314; 0.6703)	0.6581 (0.6352; 0.6747)	0.6580 (0.6445; 0.6706)
0.2	1 x 0.1	2000	0.6536 (0.6390; 0.6713)	0.6536 (0.6416; 0.6650)	0.6582 (0.6457; 0.6657)
0.2	1 x 0.1	10000	0.6551 (0.6513; 0.6609)	0.6558 (0.6494; 0.6596)	0.6556 (0.6509; 0.6587)
0.2	1 x 0.2	200	0.6901 (0.6580; 0.7326)	0.7116 (0.6757; 0.7440)	0.7150 (0.6913; 0.7418)
0.2	1 x 0.2	1000	0.7146 (0.6931; 0.7288)	0.7174 (0.7040; 0.7268)	0.7193 (0.7033; 0.7294)
0.2	1 x 0.2	2000	0.7163 (0.7031; 0.7273)	0.7150 (0.7076; 0.7273)	0.7190 (0.7105; 0.7307)
0.2	1 x 0.2	10000	0.7179 (0.7123; 0.7230)	0.7167 (0.7113; 0.7222)	0.7186 (0.7134; 0.7224)
0.4	1 x 0.05	200	0.5234 (0.4751; 0.5776)	0.5460 (0.4984; 0.5871)	0.5000 (0.5000; 0.5844)
0.4	1 x 0.05	1000	0.6059 (0.5879; 0.6280)	0.6038 (0.5902; 0.6202)	0.6190 (0.6037; 0.6339)
0.4	1 x 0.05	2000	0.6172 (0.6027; 0.6301)	0.6145 (0.5981; 0.6295)	0.6212 (0.6088; 0.6349)
0.4	1 x 0.05	10000	0.6249 (0.6182; 0.6293)	0.6235 (0.6180; 0.6275)	0.6261 (0.6192; 0.6293)
0.4	1 x 0.1	200	0.6046 (0.5518; 0.6644)	0.6350 (0.5636; 0.6722)	0.6428 (0.5554; 0.6880)
0.4	1 x 0.1	1000	0.6743 (0.6497; 0.6846)	0.6724 (0.6504; 0.6852)	0.6799 (0.6677; 0.6891)
0.4	1 x 0.1	2000	0.6799 (0.6650; 0.6916)	0.6735 (0.6643; 0.6853)	0.6802 (0.6706; 0.6895)
0.4	1 x 0.1	10000	0.6765 (0.6729; 0.6831)	0.6767 (0.6713; 0.6826)	0.6769 (0.6731; 0.6819)
0.4	1 x 0.2	200	0.6952 (0.6368; 0.7480)	0.7202 (0.6901; 0.7531)	0.7468 (0.7120; 0.7661)
0.4	1 x 0.2	1000	0.7337 (0.7115; 0.7546)	0.7323 (0.7150; 0.7520)	0.7420 (0.7242; 0.7589)
0.4	1 x 0.2	2000	0.7476 (0.7343; 0.7570)	0.7445 (0.7357; 0.7573)	0.7527 (0.7433; 0.7615)
0.4	1 x 0.2	10000	0.7487 (0.7430; 0.7523)	0.7488 (0.7417; 0.7526)	0.7480 (0.7435; 0.7520)

Performance of the algorithms `MBMDRC`, `RANGER`, and `GLMNET` measured as AUC over 50 replicates in scenario 1. The median of the AUC and the 25% and 75% quantile in parentheses over 50 replicates are given.
