# Supplementary material for: Empowering individual trait prediction using interactions for precision medicine
Source: BMC Bioinformatics. 2021 Feb 18;22:74. doi: 10.1186/s12859-021-04011-z (PMC7890638; doi:10.1186/s12859-021-04011-z)
Supplement: Supplementary file 5 — Additional file 5: Table 2. Performance in scenario 2. Performance of the algorithms MBMDRC, RANGER, and GLMNET measured as AUC over 50 replicates in scenario 2: five SNPs with main effects (MAF 0.1, 0.2, or 0.4 and heritability 0.05, 0.1, 0.2), 95 SNPs without any effect.. The median of the AUC and the 25% and 75% quantile in parentheses over 50 replicates are given. [file 12859_2021_4011_MOESM5_ESM.pdf]

Table 9 Performance in scenario 2.

MAF	h^2	n	MBMDRC	RANGER	GLMNET
0.1	5 x 0.05	200	0.5504 (0.4952; 0.6008)	0.6032 (0.5427; 0.6428)	0.5608 (0.5000; 0.6338)
0.1	5 x 0.05	1000	0.7132 (0.6941; 0.7381)	0.7183 (0.6984; 0.7337)	0.7166 (0.6933; 0.7356)
0.1	5 x 0.05	2000	0.7338 (0.7221; 0.7437)	0.7248 (0.7159; 0.7383)	0.7280 (0.7170; 0.7432)
0.1	5 x 0.05	10000	0.7361 (0.7306; 0.7397)	0.7323 (0.7262; 0.7361)	0.7343 (0.7301; 0.7393)
0.1	5 x 0.1	200	0.6400 (0.5706; 0.7052)	0.6990 (0.6516; 0.7354)	0.6558 (0.5936; 0.7220)
0.1	5 x 0.1	1000	0.8098 (0.7952; 0.8277)	0.7965 (0.7761; 0.8168)	0.8012 (0.7841; 0.8234)
0.1	5 x 0.1	2000	0.8101 (0.8022; 0.8191)	0.8037 (0.7943; 0.8110)	0.8077 (0.8002; 0.8156)
0.1	5 x 0.1	10000	0.8164 (0.8109; 0.8197)	0.8127 (0.8096; 0.8165)	0.8160 (0.8113; 0.8193)
0.1	5 x 0.2	200	0.7818 (0.7249; 0.8383)	0.8032 (0.7649; 0.8503)	0.8092 (0.7656; 0.8645)
0.1	5 x 0.2	1000	0.8907 (0.8817; 0.9011)	0.8830 (0.8732; 0.8915)	0.8920 (0.8817; 0.9006)
0.1	5 x 0.2	2000	0.8929 (0.8862; 0.8974)	0.8848 (0.8796; 0.8915)	0.8916 (0.8866; 0.8977)
0.1	5 x 0.2	10000	0.8969 (0.8944; 0.8999)	0.8936 (0.8916; 0.8957)	0.8947 (0.8916; 0.8981)
0.2	5 x 0.05	200	0.5786 (0.5248; 0.6410)	0.6094 (0.5603; 0.6765)	0.5880 (0.5000; 0.6302)
0.2	5 x 0.05	1000	0.7303 (0.7053; 0.7520)	0.7134 (0.6944; 0.7341)	0.7311 (0.7041; 0.7517)
0.2	5 x 0.05	2000	0.7421 (0.7257; 0.7562)	0.7258 (0.7125; 0.7389)	0.7357 (0.7218; 0.7475)
0.2	5 x 0.05	10000	0.7457 (0.7412; 0.7501)	0.7402 (0.7340; 0.7445)	0.7444 (0.7387; 0.7490)
0.2	5 x 0.1	200	0.6816 (0.6122; 0.7511)	0.7038 (0.6554; 0.7563)	0.6934 (0.6345; 0.7425)
0.2	5 x 0.1	1000	0.8146 (0.7990; 0.8233)	0.7969 (0.7791; 0.8044)	0.8019 (0.7898; 0.8193)
0.2	5 x 0.1	2000	0.8105 (0.8018; 0.8221)	0.7985 (0.7926; 0.8095)	0.8078 (0.8002; 0.8188)
0.2	5 x 0.1	10000	0.8140 (0.8098; 0.8177)	0.8092 (0.8037; 0.8132)	0.8131 (0.8077; 0.8164)
0.2	5 x 0.2	200	0.7663 (0.7160; 0.8126)	0.7884 (0.7371; 0.8228)	0.8061 (0.7320; 0.8504)
0.2	5 x 0.2	1000	0.8772 (0.8655; 0.8870)	0.8595 (0.8497; 0.8741)	0.8744 (0.8602; 0.8858)
0.2	5 x 0.2	2000	0.8777 (0.8718; 0.8860)	0.8675 (0.8608; 0.8741)	0.8755 (0.8673; 0.8818)
0.2	5 x 0.2	10000	0.8814 (0.8786; 0.8845)	0.8761 (0.8736; 0.8788)	0.8785 (0.8757; 0.8817)
0.4	5 x 0.05	200	0.5946 (0.5553; 0.6322)	0.6424 (0.5873; 0.6855)	0.6445 (0.5635; 0.6894)
0.4	5 x 0.05	1000	0.7510 (0.7273; 0.7660)	0.7323 (0.7162; 0.7458)	0.7504 (0.7269; 0.7624)
0.4	5 x 0.05	2000	0.7609 (0.7499; 0.7726)	0.7449 (0.7367; 0.7528)	0.7560 (0.7475; 0.7681)
0.4	5 x 0.05	10000	0.7670 (0.7615; 0.7713)	0.7591 (0.7532; 0.7642)	0.7638 (0.7586; 0.7694)
0.4	5 x 0.1	200	0.6922 (0.6408; 0.7463)	0.7362 (0.6795; 0.7702)	0.7177 (0.6828; 0.7872)
0.4	5 x 0.1	1000	0.8449 (0.8278; 0.8583)	0.8257 (0.8059; 0.8377)	0.8384 (0.8243; 0.8518)
0.4	5 x 0.1	2000	0.8446 (0.8372; 0.8533)	0.8298 (0.8231; 0.8380)	0.8393 (0.8317; 0.8471)
0.4	5 x 0.1	10000	0.8474 (0.8424; 0.8502)	0.8412 (0.8368; 0.8440)	0.8459 (0.8422; 0.8490)
0.4	5 x 0.2	200	0.8122 (0.7662; 0.8564)	0.8390 (0.8094; 0.8668)	0.8415 (0.7967; 0.8766)
0.4	5 x 0.2	1000	0.9177 (0.9077; 0.9269)	0.9003 (0.8935; 0.9118)	0.9121 (0.9026; 0.9213)
0.4	5 x 0.2	2000	0.9169 (0.9124; 0.9223)	0.9070 (0.9027; 0.9111)	0.9130 (0.9082; 0.9176)
0.4	5 x 0.2	10000	0.9166 (0.9141; 0.9189)	0.9122 (0.9095; 0.9149)	0.9129 (0.9110; 0.9155)

Performance of the algorithms `MBMDRC`, `RANGER`, and `GLMNET` measured as AUC over 50 replicates in scenario 2. The median of the AUC and the 25% and 75% quantile in parentheses over 50 replicates are given.
