# Supplementary material for: Empowering individual trait prediction using interactions for precision medicine
Source: BMC Bioinformatics. 2021 Feb 18;22:74. doi: 10.1186/s12859-021-04011-z (PMC7890638; doi:10.1186/s12859-021-04011-z)
Supplement: Supplementary file 6 — Additional file 6: Table 3. Performance in scenario 3. Performance of the algorithms MBMDRC, RANGER, and GLMNET measured as AUC over 50 replicates in scenario 3: one pair of interacting SNPs without marginal effects (MAF 0.1, 0.2, or 0.4 and heritability 0.05, 0.1, 0.2), 98 SNPs without any effect. The median of the AUC and the 25% and 75% quantile in parentheses over 50 replicates are given. [file 12859_2021_4011_MOESM6_ESM.pdf]

Table 10 Performance in scenario 3.

MAF	h^2	n	MBMDRC	RANGER	GLMNET
0.1,0.1	1 x 0.05	200	0.5355 (0.4856; 0.5861)	0.4996 (0.4543; 0.5375)	0.5000 (0.5000; 0.5268)
0.1,0.1	1 x 0.05	1000	0.5430 (0.5101; 0.5836)	0.5017 (0.4837; 0.5189)	0.5000 (0.4961; 0.5036)
0.1,0.1	1 x 0.05	2000	0.5796 (0.5647; 0.5929)	0.5018 (0.4866; 0.5139)	0.5000 (0.4960; 0.5013)
0.1,0.1	1 x 0.05	10000	0.5886 (0.5835; 0.5950)	0.5196 (0.5143; 0.5283)	0.5000 (0.4972; 0.5000)
0.1,0.1	1 x 0.1	200	0.5020 (0.4685; 0.5432)	0.4914 (0.4557; 0.5154)	0.5000 (0.4873; 0.5000)
0.1,0.1	1 x 0.1	1000	0.6523 (0.6397; 0.6703)	0.5138 (0.4987; 0.5377)	0.5000 (0.4984; 0.5007)
0.1,0.1	1 x 0.1	2000	0.6649 (0.6566; 0.6767)	0.5194 (0.5045; 0.5375)	0.5000 (0.4937; 0.5000)
0.1,0.1	1 x 0.1	10000	0.6671 (0.6587; 0.6749)	0.5601 (0.5515; 0.5735)	0.5000 (0.4980; 0.5015)
0.1,0.1	1 x 0.2	200	0.5602 (0.4945; 0.6581)	0.4934 (0.4436; 0.5268)	0.5000 (0.4702; 0.5000)
0.1,0.1	1 x 0.2	1000	0.7248 (0.7113; 0.7352)	0.5326 (0.5089; 0.5572)	0.5000 (0.5000; 0.5128)
0.1,0.1	1 x 0.2	2000	0.7230 (0.7168; 0.7318)	0.5507 (0.5270; 0.5787)	0.5000 (0.4851; 0.5000)
0.1,0.1	1 x 0.2	10000	0.7253 (0.7210; 0.7293)	0.6316 (0.6142; 0.6507)	0.5000 (0.5000; 0.5033)
0.1,0.2	1 x 0.05	200	0.5100 (0.4689; 0.5698)	0.4946 (0.4587; 0.5557)	0.5000 (0.4814; 0.5158)
0.1,0.2	1 x 0.05	1000	0.5883 (0.5271; 0.6152)	0.5282 (0.5036; 0.5472)	0.5000 (0.5000; 0.5201)
0.1,0.2	1 x 0.05	2000	0.6178 (0.6069; 0.6287)	0.5555 (0.5237; 0.5798)	0.5000 (0.5000; 0.5261)
0.1,0.2	1 x 0.05	10000	0.6249 (0.6168; 0.6348)	0.6201 (0.6119; 0.6314)	0.5396 (0.5290; 0.5619)
0.1,0.2	1 x 0.1	200	0.5180 (0.4806; 0.5636)	0.5046 (0.4530; 0.5420)	0.5000 (0.5000; 0.5258)
0.1,0.2	1 x 0.1	1000	0.6678 (0.6486; 0.6880)	0.5789 (0.5329; 0.6176)	0.5106 (0.5000; 0.5405)
0.1,0.2	1 x 0.1	2000	0.6701 (0.6618; 0.6798)	0.6483 (0.6285; 0.6592)	0.5336 (0.5000; 0.5533)
0.1,0.2	1 x 0.1	10000	0.6735 (0.6680; 0.6822)	0.6728 (0.6658; 0.6806)	0.5962 (0.5635; 0.6042)
0.1,0.2	1 x 0.2	200	0.6561 (0.5819; 0.7335)	0.5410 (0.4758; 0.6090)	0.5000 (0.4933; 0.5448)
0.1,0.2	1 x 0.2	1000	0.7685 (0.7556; 0.7814)	0.7514 (0.7326; 0.7740)	0.5821 (0.5509; 0.6194)
0.1,0.2	1 x 0.2	2000	0.7692 (0.7619; 0.7759)	0.7695 (0.7604; 0.7781)	0.6064 (0.5807; 0.6561)
0.1,0.2	1 x 0.2	10000	0.7753 (0.7690; 0.7795)	0.7750 (0.7709; 0.7800)	0.6654 (0.6556; 0.6761)
0.1,0.4	1 x 0.05	200	0.5533 (0.5023; 0.6112)	0.5204 (0.4889; 0.5752)	0.5000 (0.4884; 0.5300)
0.1,0.4	1 x 0.05	1000	0.6268 (0.5383; 0.6830)	0.5767 (0.5305; 0.6662)	0.5214 (0.5000; 0.5548)
0.1,0.4	1 x 0.05	2000	0.6520 (0.5901; 0.6871)	0.6315 (0.5621; 0.6824)	0.5443 (0.5005; 0.5982)
0.1,0.4	1 x 0.05	10000	0.6643 (0.6044; 0.6912)	0.6554 (0.5951; 0.6875)	0.5595 (0.5370; 0.6192)
0.1,0.4	1 x 0.1	200	0.6928 (0.5658; 0.7620)	0.5996 (0.5443; 0.6825)	0.5000 (0.4941; 0.5454)
0.1,0.4	1 x 0.1	1000	0.7665 (0.7095; 0.8050)	0.7559 (0.6752; 0.7977)	0.5700 (0.5343; 0.6364)
0.1,0.4	1 x 0.1	2000	0.7903 (0.6991; 0.8132)	0.7776 (0.6919; 0.8066)	0.5900 (0.5570; 0.6695)
0.1,0.4	1 x 0.1	10000	0.7965 (0.7081; 0.8158)	0.7892 (0.7071; 0.8119)	0.6205 (0.5674; 0.6738)
0.1,0.4	1 x 0.2	200	0.8393 (0.8002; 0.8731)	0.7616 (0.6871; 0.8072)	0.5478 (0.5000; 0.5964)
0.1,0.4	1 x 0.2	1000	0.8626 (0.8381; 0.8793)	0.8427 (0.8320; 0.8665)	0.6039 (0.5832; 0.6559)
0.1,0.4	1 x 0.2	2000	0.8540 (0.8460; 0.8846)	0.8555 (0.8426; 0.8745)	0.6597 (0.6041; 0.7034)
0.1,0.4	1 x 0.2	10000	0.8522 (0.8447; 0.8810)	0.8516 (0.8421; 0.8774)	0.6703 (0.6342; 0.7087)
0.2,0.2	1 x 0.05	200	0.5036 (0.4561; 0.5484)	0.4944 (0.4529; 0.5404)	0.5000 (0.4896; 0.5173)
0.2,0.2	1 x 0.05	1000	0.5494 (0.5054; 0.5927)	0.5007 (0.4851; 0.5155)	0.5000 (0.4951; 0.5048)
0.2,0.2	1 x 0.05	2000	0.6084 (0.5931; 0.6228)	0.4982 (0.4867; 0.5133)	0.5000 (0.4921; 0.5000)
0.2,0.2	1 x 0.05	10000	0.6146 (0.6101; 0.6206)	0.5192 (0.5109; 0.5317)	0.5000 (0.4967; 0.5000)
0.2,0.2	1 x 0.1	200	0.5116 (0.4673; 0.5753)	0.4938 (0.4588; 0.5414)	0.5000 (0.4824; 0.5070)
0.2,0.2	1 x 0.1	1000	0.6568 (0.6373; 0.6721)	0.5029 (0.4874; 0.5209)	0.5000 (0.4961; 0.5030)

0.2,0.2	1 x 0.1	2000	0.6637 (0.6498; 0.6731)	0.5092 (0.4949; 0.5275)	0.5000 (0.4919; 0.5037)
0.2,0.2	1 x 0.1	10000	0.6644 (0.6605; 0.6685)	0.5434 (0.5310; 0.5678)	0.5000 (0.4967; 0.5000)
0.2,0.2	1 x 0.2	200	0.5590 (0.5086; 0.6306)	0.4966 (0.4506; 0.5392)	0.5000 (0.5000; 0.5136)
0.2,0.2	1 x 0.2	1000	0.7264 (0.7098; 0.7381)	0.5164 (0.4948; 0.5376)	0.5000 (0.4920; 0.5047)
0.2,0.2	1 x 0.2	2000	0.7285 (0.7168; 0.7442)	0.5343 (0.5132; 0.5570)	0.5000 (0.4935; 0.5000)
0.2,0.2	1 x 0.2	10000	0.7326 (0.7279; 0.7389)	0.5983 (0.5839; 0.6260)	0.5000 (0.5000; 0.5000)
0.2,0.4	1 x 0.05	200	0.5276 (0.4668; 0.5744)	0.5098 (0.4609; 0.5491)	0.5000 (0.5000; 0.5328)
0.2,0.4	1 x 0.05	1000	0.5835 (0.5269; 0.6374)	0.5328 (0.5086; 0.5781)	0.5013 (0.5000; 0.5391)
0.2,0.4	1 x 0.05	2000	0.6311 (0.5739; 0.6651)	0.5808 (0.5075; 0.6223)	0.5080 (0.5000; 0.5610)
0.2,0.4	1 x 0.05	10000	0.6398 (0.5932; 0.6544)	0.6282 (0.5554; 0.6552)	0.5261 (0.5000; 0.5855)
0.2,0.4	1 x 0.1	200	0.5256 (0.4872; 0.5790)	0.5104 (0.4678; 0.5663)	0.5000 (0.4922; 0.5268)
0.2,0.4	1 x 0.1	1000	0.6491 (0.5827; 0.7144)	0.5839 (0.5063; 0.6538)	0.5000 (0.4978; 0.5203)
0.2,0.4	1 x 0.1	2000	0.6742 (0.6370; 0.7206)	0.6481 (0.5373; 0.7022)	0.5044 (0.5000; 0.5515)
0.2,0.4	1 x 0.1	10000	0.6879 (0.6431; 0.7219)	0.6822 (0.6304; 0.7176)	0.5330 (0.5068; 0.5913)
0.2,0.4	1 x 0.2	200	0.7010 (0.5708; 0.7922)	0.5684 (0.5155; 0.6415)	0.5147 (0.5000; 0.5636)
0.2,0.4	1 x 0.2	1000	0.7787 (0.7506; 0.8096)	0.7704 (0.6835; 0.8070)	0.5463 (0.5001; 0.6314)
0.2,0.4	1 x 0.2	2000	0.7932 (0.7400; 0.8113)	0.7866 (0.7200; 0.7985)	0.5711 (0.5292; 0.6756)
0.2,0.4	1 x 0.2	10000	0.7964 (0.7518; 0.8049)	0.7946 (0.7512; 0.8034)	0.6152 (0.5583; 0.6692)
0.4,0.4	1 x 0.05	200	0.5189 (0.4813; 0.5589)	0.4928 (0.4548; 0.5414)	0.5000 (0.4896; 0.5112)
0.4,0.4	1 x 0.05	1000	0.5472 (0.5195; 0.5941)	0.4983 (0.4830; 0.5140)	0.5000 (0.4937; 0.5015)
0.4,0.4	1 x 0.05	2000	0.6138 (0.5950; 0.6294)	0.4999 (0.4889; 0.5143)	0.5000 (0.5000; 0.5027)
0.4,0.4	1 x 0.05	10000	0.6206 (0.6170; 0.6260)	0.5165 (0.5100; 0.5265)	0.5000 (0.5000; 0.5000)
0.4,0.4	1 x 0.1	200	0.5176 (0.4690; 0.5543)	0.4828 (0.4570; 0.5369)	0.5000 (0.4943; 0.5260)
0.4,0.4	1 x 0.1	1000	0.6662 (0.6480; 0.6888)	0.5041 (0.4872; 0.5225)	0.5000 (0.4952; 0.5046)
0.4,0.4	1 x 0.1	2000	0.6708 (0.6606; 0.6804)	0.5073 (0.4931; 0.5241)	0.5000 (0.4918; 0.5003)
0.4,0.4	1 x 0.1	10000	0.6730 (0.6656; 0.6797)	0.5472 (0.5362; 0.5626)	0.5000 (0.4972; 0.5000)
0.4,0.4	1 x 0.2	200	0.5666 (0.5070; 0.6566)	0.5026 (0.4508; 0.5461)	0.5000 (0.4864; 0.5258)
0.4,0.4	1 x 0.2	1000	0.7412 (0.7312; 0.7508)	0.5133 (0.4943; 0.5280)	0.5000 (0.4965; 0.5008)
0.4,0.4	1 x 0.2	2000	0.7477 (0.7371; 0.7591)	0.5338 (0.5135; 0.5514)	0.5000 (0.4930; 0.5029)
0.4,0.4	1 x 0.2	10000	0.7479 (0.7414; 0.7528)	0.6198 (0.5903; 0.6565)	0.5000 (0.4968; 0.5000)

Performance of the algorithms `MBMDRC`, `RANGER`, and `GLMNET` measured as AUC over 50 replicates in scenario 3. The median of the AUC and the 25% and 75% quantile in parentheses over 50 replicates are given.
