# Supplementary material for: Empowering individual trait prediction using interactions for precision medicine
Source: BMC Bioinformatics. 2021 Feb 18;22:74. doi: 10.1186/s12859-021-04011-z (PMC7890638; doi:10.1186/s12859-021-04011-z)
Supplement: Supplementary file 7 — Additional file 7: Table 4. Performance in scenario 4. Performance of the algorithms MBMDRC, RANGER, and GLMNET measured as AUC over 50 replicates in scenario 4: one pair of interacting SNPs without marginal effects and three SNPs with main effects (MAF 0.1, 0.2, or 0.4 and heritability 0.05, 0.1, 0.2), 95 SNPs without any effect.. The median of the AUC and the 25% and 75% quantile in parentheses over 50 replicates are given. [file 12859_2021_4011_MOESM7_ESM.pdf]

Table 11 Performance in scenario 4.

MAF	h^2	n	MBMDRC	RANGER	GLMNET
0.1,0.1; 0.1; 0.2; 0.4	4 x 0.05	200	0.5415 (0.4867; 0.6123)	0.5702 (0.5132; 0.6059)	0.5381 (0.5000; 0.5929)
0.1,0.1; 0.1; 0.2; 0.4	4 x 0.05	1000	0.6729 (0.6467; 0.6989)	0.6593 (0.6444; 0.6807)	0.6721 (0.6402; 0.6951)
0.1,0.1; 0.1; 0.2; 0.4	4 x 0.05	2000	0.7120 (0.6948; 0.7316)	0.6813 (0.6709; 0.6968)	0.6812 (0.6667; 0.6956)
0.1,0.1; 0.1; 0.2; 0.4	4 x 0.05	10000	0.7311 (0.7234; 0.7400)	0.7049 (0.6949; 0.7203)	0.6903 (0.6794; 0.6993)
0.1,0.1; 0.1; 0.2; 0.4	4 x 0.1	200	0.5910 (0.5397; 0.6520)	0.6496 (0.5891; 0.6943)	0.6474 (0.5801; 0.6945)
0.1,0.1; 0.1; 0.2; 0.4	4 x 0.1	1000	0.7910 (0.7737; 0.8082)	0.7356 (0.7146; 0.7492)	0.7339 (0.7128; 0.7587)
0.1,0.1; 0.1; 0.2; 0.4	4 x 0.1	2000	0.8102 (0.7947; 0.8239)	0.7484 (0.7338; 0.7629)	0.7460 (0.7327; 0.7575)
0.1,0.1; 0.1; 0.2; 0.4	4 x 0.1	10000	0.8170 (0.8112; 0.8215)	0.7792 (0.7643; 0.7951)	0.7545 (0.7446; 0.7666)
0.1,0.1; 0.1; 0.2; 0.4	4 x 0.2	200	0.6695 (0.6109; 0.7443)	0.7000 (0.6563; 0.7300)	0.7099 (0.6570; 0.7498)
0.1,0.1; 0.1; 0.2; 0.4	4 x 0.2	1000	0.8804 (0.8666; 0.8951)	0.7982 (0.7845; 0.8179)	0.7706 (0.7565; 0.7894)
0.1,0.1; 0.1; 0.2; 0.4	4 x 0.2	2000	0.8907 (0.8819; 0.8991)	0.8279 (0.8171; 0.8427)	0.7823 (0.7693; 0.7952)
0.1,0.1; 0.1; 0.2; 0.4	4 x 0.2	10000	0.8938 (0.8899; 0.8974)	0.8756 (0.8724; 0.8797)	0.7968 (0.7840; 0.8081)
0.1,0.2; 0.1; 0.2; 0.4	4 x 0.05	200	0.5612 (0.5042; 0.6228)	0.5844 (0.5241; 0.6302)	0.5520 (0.5000; 0.6397)
0.1,0.2; 0.1; 0.2; 0.4	4 x 0.05	1000	0.6700 (0.6440; 0.6997)	0.6676 (0.6541; 0.6902)	0.6639 (0.6480; 0.6888)
0.1,0.2; 0.1; 0.2; 0.4	4 x 0.05	2000	0.7205 (0.7057; 0.7345)	0.6819 (0.6697; 0.6914)	0.6819 (0.6701; 0.6938)
0.1,0.2; 0.1; 0.2; 0.4	4 x 0.05	10000	0.7385 (0.7308; 0.7439)	0.7134 (0.7097; 0.7194)	0.6963 (0.6918; 0.7013)
0.1,0.2; 0.1; 0.2; 0.4	4 x 0.1	200	0.5800 (0.5299; 0.6376)	0.6242 (0.5934; 0.6695)	0.6096 (0.5438; 0.6592)
0.1,0.2; 0.1; 0.2; 0.4	4 x 0.1	1000	0.7678 (0.7406; 0.7917)	0.7369 (0.7173; 0.7541)	0.7324 (0.7077; 0.7439)
0.1,0.2; 0.1; 0.2; 0.4	4 x 0.1	2000	0.8004 (0.7911; 0.8088)	0.7541 (0.7373; 0.7640)	0.7372 (0.7235; 0.7511)
0.1,0.2; 0.1; 0.2; 0.4	4 x 0.1	10000	0.8141 (0.8094; 0.8169)	0.7961 (0.7914; 0.8008)	0.7551 (0.7420; 0.7618)
0.1,0.2; 0.1; 0.2; 0.4	4 x 0.2	200	0.6662 (0.6113; 0.7121)	0.6990 (0.6601; 0.7396)	0.6706 (0.6248; 0.7082)
0.1,0.2; 0.1; 0.2; 0.4	4 x 0.2	1000	0.8718 (0.8469; 0.8897)	0.8458 (0.8275; 0.8641)	0.7793 (0.7616; 0.7960)
0.1,0.2; 0.1; 0.2; 0.4	4 x 0.2	2000	0.8866 (0.8768; 0.8947)	0.8667 (0.8583; 0.8739)	0.7859 (0.7719; 0.7992)
0.1,0.2; 0.1; 0.2; 0.4	4 x 0.2	10000	0.8977 (0.8885; 0.9048)	0.8959 (0.8899; 0.8992)	0.8006 (0.7955; 0.8056)
0.1,0.4; 0.1; 0.2; 0.4	4 x 0.05	200	0.5588 (0.5146; 0.6119)	0.5904 (0.5352; 0.6211)	0.5505 (0.5000; 0.6036)
0.1,0.4; 0.1; 0.2; 0.4	4 x 0.05	1000	0.6971 (0.6598; 0.7249)	0.6853 (0.6621; 0.7238)	0.6690 (0.6491; 0.6985)
0.1,0.4; 0.1; 0.2; 0.4	4 x 0.05	2000	0.7180 (0.6913; 0.7446)	0.7073 (0.6798; 0.7395)	0.6903 (0.6701; 0.7087)
0.1,0.4; 0.1; 0.2; 0.4	4 x 0.05	10000	0.7400 (0.7261; 0.7585)	0.7332 (0.7082; 0.7617)	0.7012 (0.6955; 0.7108)
0.1,0.4; 0.1; 0.2; 0.4	4 x 0.1	200	0.6622 (0.5951; 0.7451)	0.6802 (0.6234; 0.7334)	0.6096 (0.5602; 0.6788)
0.1,0.4; 0.1; 0.2; 0.4	4 x 0.1	1000	0.8098 (0.7713; 0.8275)	0.7965 (0.7463; 0.8326)	0.7172 (0.6934; 0.7405)
0.1,0.4; 0.1; 0.2; 0.4	4 x 0.1	2000	0.8275 (0.8029; 0.8498)	0.8344 (0.7741; 0.8490)	0.7346 (0.7124; 0.7621)
0.1,0.4; 0.1; 0.2; 0.4	4 x 0.1	10000	0.8457 (0.8181; 0.8560)	0.8537 (0.8031; 0.8673)	0.7537 (0.7407; 0.7677)
0.1,0.4; 0.1; 0.2; 0.4	4 x 0.2	200	0.7464 (0.6386; 0.8187)	0.7430 (0.6756; 0.7844)	0.6224 (0.5270; 0.6916)
0.1,0.4; 0.1; 0.2; 0.4	4 x 0.2	1000	0.8666 (0.8524; 0.8779)	0.8693 (0.8493; 0.8928)	0.7602 (0.7327; 0.7815)
0.1,0.4; 0.1; 0.2; 0.4	4 x 0.2	2000	0.8827 (0.8711; 0.9003)	0.8919 (0.8774; 0.9099)	0.7701 (0.7496; 0.7959)
0.1,0.4; 0.1; 0.2; 0.4	4 x 0.2	10000	0.8934 (0.8811; 0.9086)	0.9103 (0.9022; 0.9258)	0.7858 (0.7679; 0.8096)
0.2,0.2; 0.1; 0.2; 0.4	4 x 0.05	200	0.5398 (0.5059; 0.6000)	0.5794 (0.5337; 0.6174)	0.5448 (0.5000; 0.5902)
0.2,0.2; 0.1; 0.2; 0.4	4 x 0.05	1000	0.6767 (0.6478; 0.6935)	0.6671 (0.6494; 0.6848)	0.6744 (0.6538; 0.6955)
0.2,0.2; 0.1; 0.2; 0.4	4 x 0.05	2000	0.7075 (0.6964; 0.7254)	0.6718 (0.6637; 0.6891)	0.6816 (0.6666; 0.6922)
0.2,0.2; 0.1; 0.2; 0.4	4 x 0.05	10000	0.7301 (0.7255; 0.7358)	0.6942 (0.6883; 0.6991)	0.6923 (0.6855; 0.6977)
0.2,0.2; 0.1; 0.2; 0.4	4 x 0.1	200	0.6093 (0.5340; 0.6759)	0.6692 (0.6040; 0.7203)	0.6424 (0.5708; 0.7168)
0.2,0.2; 0.1; 0.2; 0.4	4 x 0.1	1000	0.7627 (0.7378; 0.7827)	0.7317 (0.7120; 0.7504)	0.7324 (0.7123; 0.7549)
0.2,0.2; 0.1; 0.2; 0.4	4 x 0.1	2000	0.7906 (0.7803; 0.8003)	0.7394 (0.7263; 0.7511)	0.7440 (0.7317; 0.7537)
0.2,0.2; 0.1; 0.2; 0.4	4 x 0.1	10000	0.8022 (0.7978; 0.8050)	0.7586 (0.7535; 0.7634)	0.7548 (0.7494; 0.7590)
0.2,0.2; 0.1; 0.2; 0.4	4 x 0.2	200	0.6518 (0.5838; 0.7064)	0.7132 (0.6752; 0.7579)	0.7122 (0.6432; 0.7566)
0.2,0.2; 0.1; 0.2; 0.4	4 x 0.2	1000	0.8589 (0.8474; 0.8758)	0.7848 (0.7721; 0.7973)	0.7889 (0.7722; 0.8031)

0.2,0.2; 0.1; 0.2; 0.4	4 x 0.2	2000	0.8687 (0.8615; 0.8775)	0.7995 (0.7896; 0.8044)	0.7958 (0.7824; 0.8067)
0.2,0.2; 0.1; 0.2; 0.4	4 x 0.2	10000	0.8748 (0.8686; 0.8775)	0.8324 (0.8247; 0.8379)	0.8017 (0.7964; 0.8082)
0.2,0.4; 0.1; 0.2; 0.4	4 x 0.05	200	0.5485 (0.4959; 0.6187)	0.5622 (0.5302; 0.6086)	0.5356 (0.5000; 0.5648)
0.2,0.4; 0.1; 0.2; 0.4	4 x 0.05	1000	0.6730 (0.6549; 0.7021)	0.6718 (0.6504; 0.6970)	0.6638 (0.6502; 0.6945)
0.2,0.4; 0.1; 0.2; 0.4	4 x 0.05	2000	0.7070 (0.6931; 0.7312)	0.6932 (0.6767; 0.7062)	0.6876 (0.6691; 0.7006)
0.2,0.4; 0.1; 0.2; 0.4	4 x 0.05	10000	0.7338 (0.7217; 0.7506)	0.7149 (0.7027; 0.7276)	0.6998 (0.6924; 0.7067)
0.2,0.4; 0.1; 0.2; 0.4	4 x 0.1	200	0.5972 (0.5328; 0.6478)	0.6428 (0.5925; 0.6905)	0.6105 (0.5444; 0.6844)
0.2,0.4; 0.1; 0.2; 0.4	4 x 0.1	1000	0.7640 (0.7393; 0.7892)	0.7392 (0.7228; 0.7597)	0.7288 (0.7099; 0.7493)
0.2,0.4; 0.1; 0.2; 0.4	4 x 0.1	2000	0.7906 (0.7729; 0.8092)	0.7532 (0.7386; 0.7672)	0.7456 (0.7311; 0.7593)
0.2,0.4; 0.1; 0.2; 0.4	4 x 0.1	10000	0.8038 (0.7944; 0.8172)	0.7811 (0.7668; 0.7995)	0.7574 (0.7488; 0.7647)
0.2,0.4; 0.1; 0.2; 0.4	4 x 0.2	200	0.6636 (0.6176; 0.7173)	0.7106 (0.6632; 0.7603)	0.6853 (0.6446; 0.7266)
0.2,0.4; 0.1; 0.2; 0.4	4 x 0.2	1000	0.8552 (0.8346; 0.8729)	0.8159 (0.7929; 0.8411)	0.7901 (0.7713; 0.8083)
0.2,0.4; 0.1; 0.2; 0.4	4 x 0.2	2000	0.8759 (0.8553; 0.8841)	0.8342 (0.8244; 0.8535)	0.7947 (0.7828; 0.8102)
0.2,0.4; 0.1; 0.2; 0.4	4 x 0.2	10000	0.8825 (0.8698; 0.8898)	0.8735 (0.8472; 0.8791)	0.8121 (0.7990; 0.8188)
0.4,0.4; 0.1; 0.2; 0.4	4 x 0.05	200	0.5534 (0.4935; 0.6192)	0.5822 (0.5489; 0.6190)	0.5480 (0.5000; 0.6232)
0.4,0.4; 0.1; 0.2; 0.4	4 x 0.05	1000	0.6757 (0.6422; 0.6928)	0.6630 (0.6479; 0.6785)	0.6740 (0.6511; 0.6851)
0.4,0.4; 0.1; 0.2; 0.4	4 x 0.05	2000	0.7162 (0.7003; 0.7295)	0.6809 (0.6671; 0.6913)	0.6920 (0.6694; 0.7010)
0.4,0.4; 0.1; 0.2; 0.4	4 x 0.05	10000	0.7320 (0.7250; 0.7364)	0.6960 (0.6906; 0.7015)	0.6953 (0.6901; 0.7003)
0.4,0.4; 0.1; 0.2; 0.4	4 x 0.1	200	0.6058 (0.5240; 0.6708)	0.6618 (0.5994; 0.6910)	0.6451 (0.5624; 0.6961)
0.4,0.4; 0.1; 0.2; 0.4	4 x 0.1	1000	0.7571 (0.7408; 0.7723)	0.7289 (0.7162; 0.7485)	0.7422 (0.7211; 0.7544)
0.4,0.4; 0.1; 0.2; 0.4	4 x 0.1	2000	0.7931 (0.7813; 0.8027)	0.7391 (0.7249; 0.7485)	0.7444 (0.7257; 0.7535)
0.4,0.4; 0.1; 0.2; 0.4	4 x 0.1	10000	0.8006 (0.7974; 0.8045)	0.7555 (0.7509; 0.7606)	0.7535 (0.7505; 0.7595)
0.4,0.4; 0.1; 0.2; 0.4	4 x 0.2	200	0.6672 (0.6100; 0.7296)	0.7258 (0.6947; 0.7662)	0.7384 (0.6855; 0.7706)
0.4,0.4; 0.1; 0.2; 0.4	4 x 0.2	1000	0.8550 (0.8377; 0.8672)	0.7916 (0.7710; 0.8076)	0.7904 (0.7740; 0.8081)
0.4,0.4; 0.1; 0.2; 0.4	4 x 0.2	2000	0.8652 (0.8586; 0.8720)	0.7954 (0.7862; 0.8037)	0.7962 (0.7791; 0.8069)
0.4,0.4; 0.1; 0.2; 0.4	4 x 0.2	10000	0.8724 (0.8687; 0.8768)	0.8176 (0.8134; 0.8238)	0.8061 (0.8016; 0.8114)

Performance of the algorithms `MBMDRC`, `RANGER`, and `GLMNET` measured as AUC over 50 replicates in scenario 4. The median of the AUC and the 25% and 75% quantile in parentheses over 50 replicates are given.
