# Supplementary material for: Empowering individual trait prediction using interactions for precision medicine
Source: BMC Bioinformatics. 2021 Feb 18;22:74. doi: 10.1186/s12859-021-04011-z (PMC7890638; doi:10.1186/s12859-021-04011-z)
Supplement: Supplementary file 8 — Additional file 8: Table 5. Performance in scenario 5. Performance of the algorithms MBMDRC, RANGER, and GLMNET measured as AUC over 50 replicates in scenario 5: two pairs of interacting SNPs without marginal effects (MAF 0.1, 0.2, or 0.4 and heritability 0.05, 0.1, 0.2), 96 SNPs without any effect.. The median of the AUC and the 25% and 75% quantile in parentheses over 50 replicates are given. [file 12859_2021_4011_MOESM8_ESM.pdf]

Table 12 Performance in scenario 5.

MAF	h^2	n	MBMDRC	RANGER	GLMNET
0.1,0.1; 0.1,0.1	2 x 0.05	200	0.5018 (0.4482; 0.5447)	0.4742 (0.4370; 0.5245)	0.5000 (0.4776; 0.5076)
0.1,0.1; 0.1,0.1	2 x 0.05	1000	0.5729 (0.5381; 0.6217)	0.5078 (0.4883; 0.5334)	0.5000 (0.4995; 0.5050)
0.1,0.1; 0.1,0.1	2 x 0.05	2000	0.6341 (0.6177; 0.6443)	0.5091 (0.4975; 0.5215)	0.5000 (0.4963; 0.5000)
0.1,0.1; 0.1,0.1	2 x 0.05	10000	0.6423 (0.6365; 0.6460)	0.5369 (0.5291; 0.5487)	0.5000 (0.4992; 0.5000)
0.1,0.1; 0.1,0.1	2 x 0.1	200	0.5295 (0.4705; 0.5998)	0.5036 (0.4469; 0.5540)	0.5000 (0.4847; 0.5120)
0.1,0.1; 0.1,0.1	2 x 0.1	1000	0.7614 (0.7394; 0.7796)	0.6542 (0.6093; 0.6872)	0.5380 (0.5000; 0.5683)
0.1,0.1; 0.1,0.1	2 x 0.1	2000	0.7672 (0.7431; 0.7866)	0.7312 (0.6911; 0.7515)	0.5461 (0.5123; 0.6044)
0.1,0.1; 0.1,0.1	2 x 0.1	10000	0.7690 (0.7514; 0.7873)	0.7614 (0.7486; 0.7851)	0.6103 (0.5457; 0.6428)
0.1,0.1; 0.1,0.1	2 x 0.2	200	0.6838 (0.5958; 0.7467)	0.5660 (0.5059; 0.6247)	0.5298 (0.5000; 0.5716)
0.1,0.1; 0.1,0.1	2 x 0.2	1000	0.8552 (0.8317; 0.8703)	0.8452 (0.8145; 0.8637)	0.6320 (0.6023; 0.6588)
0.1,0.1; 0.1,0.1	2 x 0.2	2000	0.8508 (0.8330; 0.8812)	0.8499 (0.8258; 0.8774)	0.6519 (0.6124; 0.6992)
0.1,0.1; 0.1,0.1	2 x 0.2	10000	0.8481 (0.8390; 0.8796)	0.8461 (0.8358; 0.8786)	0.6942 (0.6252; 0.7088)
0.1,0.1; 0.2,0.2	2 x 0.05	200	0.4951 (0.4496; 0.5640)	0.4678 (0.4320; 0.5213)	0.5000 (0.4804; 0.5000)
0.1,0.1; 0.2,0.2	2 x 0.05	1000	0.5874 (0.5502; 0.6151)	0.5051 (0.4891; 0.5286)	0.5000 (0.4985; 0.5088)
0.1,0.1; 0.2,0.2	2 x 0.05	2000	0.6502 (0.6366; 0.6650)	0.5089 (0.4969; 0.5203)	0.5000 (0.4969; 0.5000)
0.1,0.1; 0.2,0.2	2 x 0.05	10000	0.6575 (0.6531; 0.6615)	0.5362 (0.5284; 0.5494)	0.5000 (0.4972; 0.5000)
0.1,0.1; 0.2,0.2	2 x 0.1	200	0.5434 (0.4746; 0.6322)	0.4806 (0.4463; 0.5181)	0.5000 (0.4820; 0.5094)
0.1,0.1; 0.2,0.2	2 x 0.1	1000	0.7782 (0.7485; 0.7954)	0.5772 (0.5486; 0.6289)	0.5055 (0.5000; 0.5335)
0.1,0.1; 0.2,0.2	2 x 0.1	2000	0.7826 (0.7661; 0.7942)	0.6532 (0.5906; 0.6972)	0.5084 (0.5000; 0.5411)
0.1,0.1; 0.2,0.2	2 x 0.1	10000	0.7850 (0.7674; 0.7937)	0.7546 (0.7319; 0.7666)	0.5387 (0.5230; 0.5926)
0.1,0.1; 0.2,0.2	2 x 0.2	200	0.6704 (0.5762; 0.7401)	0.4882 (0.4579; 0.5323)	0.5000 (0.4676; 0.5189)
0.1,0.1; 0.2,0.2	2 x 0.2	1000	0.8537 (0.8424; 0.8643)	0.7350 (0.6601; 0.7630)	0.5339 (0.5000; 0.5626)
0.1,0.1; 0.2,0.2	2 x 0.2	2000	0.8550 (0.8463; 0.8652)	0.7872 (0.7663; 0.8166)	0.5426 (0.5159; 0.6173)
0.1,0.1; 0.2,0.2	2 x 0.2	10000	0.8585 (0.8501; 0.8642)	0.8558 (0.8455; 0.8617)	0.5507 (0.5379; 0.6327)
0.1,0.1; 0.4,0.4	2 x 0.05	200	0.4884 (0.4392; 0.5440)	0.4734 (0.4356; 0.5165)	0.5000 (0.4818; 0.5000)
0.1,0.1; 0.4,0.4	2 x 0.05	1000	0.5746 (0.5330; 0.6174)	0.5058 (0.4876; 0.5304)	0.5000 (0.4980; 0.5088)
0.1,0.1; 0.4,0.4	2 x 0.05	2000	0.6508 (0.6396; 0.6662)	0.5054 (0.4956; 0.5175)	0.5000 (0.4986; 0.5019)
0.1,0.1; 0.4,0.4	2 x 0.05	10000	0.6586 (0.6564; 0.6636)	0.5381 (0.5267; 0.5448)	0.5000 (0.4990; 0.5000)
0.1,0.1; 0.4,0.4	2 x 0.1	200	0.5120 (0.4561; 0.5588)	0.4814 (0.4441; 0.5067)	0.5000 (0.4747; 0.5022)
0.1,0.1; 0.4,0.4	2 x 0.1	1000	0.7421 (0.7240; 0.7644)	0.5350 (0.5114; 0.5658)	0.5000 (0.4966; 0.5027)
0.1,0.1; 0.4,0.4	2 x 0.1	2000	0.7511 (0.7395; 0.7616)	0.5651 (0.5417; 0.6097)	0.5000 (0.5000; 0.5145)
0.1,0.1; 0.4,0.4	2 x 0.1	10000	0.7575 (0.7499; 0.7658)	0.6944 (0.6776; 0.7229)	0.5129 (0.5000; 0.5278)
0.1,0.1; 0.4,0.4	2 x 0.2	200	0.6307 (0.5475; 0.7046)	0.4970 (0.4664; 0.5456)	0.5000 (0.4991; 0.5000)
0.1,0.1; 0.4,0.4	2 x 0.2	1000	0.8476 (0.8349; 0.8604)	0.6328 (0.5583; 0.7149)	0.5000 (0.5000; 0.5367)
0.1,0.1; 0.4,0.4	2 x 0.2	2000	0.8460 (0.8354; 0.8572)	0.6940 (0.6220; 0.7598)	0.5113 (0.5000; 0.5448)
0.1,0.1; 0.4,0.4	2 x 0.2	10000	0.8514 (0.8470; 0.8557)	0.8375 (0.8132; 0.8467)	0.5466 (0.5289; 0.5896)
0.2,0.2; 0.2,0.2	2 x 0.05	200	0.5099 (0.4680; 0.5507)	0.4626 (0.4282; 0.5151)	0.5000 (0.4733; 0.5005)
0.2,0.2; 0.2,0.2	2 x 0.05	1000	0.5775 (0.5389; 0.6184)	0.5085 (0.4900; 0.5290)	0.5000 (0.4986; 0.5109)
0.2,0.2; 0.2,0.2	2 x 0.05	2000	0.6552 (0.6359; 0.6669)	0.5055 (0.4942; 0.5143)	0.5000 (0.4972; 0.5000)
0.2,0.2; 0.2,0.2	2 x 0.05	10000	0.6631 (0.6592; 0.6696)	0.5383 (0.5255; 0.5552)	0.5000 (0.4978; 0.5000)
0.2,0.2; 0.2,0.2	2 x 0.1	200	0.5104 (0.4559; 0.5708)	0.4614 (0.4290; 0.5134)	0.5000 (0.4684; 0.5000)
0.2,0.2; 0.2,0.2	2 x 0.1	1000	0.7166 (0.6938; 0.7337)	0.5152 (0.4986; 0.5377)	0.5000 (0.5000; 0.5099)
0.2,0.2; 0.2,0.2	2 x 0.1	2000	0.7253 (0.7126; 0.7389)	0.5244 (0.5094; 0.5381)	0.5000 (0.4959; 0.5021)
0.2,0.2; 0.2,0.2	2 x 0.1	10000	0.7308 (0.7238; 0.7353)	0.5804 (0.5575; 0.5975)	0.5000 (0.4988; 0.5000)
0.2,0.2; 0.2,0.2	2 x 0.2	200	0.5273 (0.4582; 0.6190)	0.4706 (0.4218; 0.5199)	0.5000 (0.4745; 0.5000)
0.2,0.2; 0.2,0.2	2 x 0.2	1000	0.7994 (0.7873; 0.8181)	0.5352 (0.5108; 0.5650)	0.5000 (0.4970; 0.5020)

0.2,0.2; 0.2,0.2	2 x 0.2	2000	0.8103 (0.7986; 0.8203)	0.5497 (0.5291; 0.6064)	0.5000 (0.4929; 0.5000)
0.2,0.2; 0.2,0.2	2 x 0.2	10000	0.8139 (0.8098; 0.8193)	0.6950 (0.6361; 0.7439)	0.5000 (0.5000; 0.5013)
0.2,0.2; 0.4,0.4	2 x 0.05	200	0.4968 (0.4626; 0.5564)	0.4714 (0.4342; 0.5195)	0.5000 (0.4948; 0.5072)
0.2,0.2; 0.4,0.4	2 x 0.05	1000	0.5698 (0.5280; 0.6089)	0.5057 (0.4867; 0.5273)	0.5000 (0.5000; 0.5108)
0.2,0.2; 0.4,0.4	2 x 0.05	2000	0.6567 (0.6398; 0.6695)	0.5030 (0.4913; 0.5169)	0.5000 (0.4970; 0.5000)
0.2,0.2; 0.4,0.4	2 x 0.05	10000	0.6683 (0.6625; 0.6728)	0.5312 (0.5240; 0.5405)	0.5000 (0.4977; 0.5000)
0.2,0.2; 0.4,0.4	2 x 0.1	200	0.5064 (0.4639; 0.5667)	0.4700 (0.4317; 0.5068)	0.5000 (0.4735; 0.5000)
0.2,0.2; 0.4,0.4	2 x 0.1	1000	0.7116 (0.6958; 0.7333)	0.5149 (0.4927; 0.5345)	0.5000 (0.5000; 0.5086)
0.2,0.2; 0.4,0.4	2 x 0.1	2000	0.7294 (0.7194; 0.7409)	0.5172 (0.5066; 0.5295)	0.5000 (0.4951; 0.5022)
0.2,0.2; 0.4,0.4	2 x 0.1	10000	0.7352 (0.7308; 0.7401)	0.5718 (0.5553; 0.5941)	0.5000 (0.4975; 0.5003)
0.2,0.2; 0.4,0.4	2 x 0.2	200	0.5403 (0.4834; 0.6339)	0.4738 (0.4309; 0.5153)	0.5000 (0.4612; 0.5000)
0.2,0.2; 0.4,0.4	2 x 0.2	1000	0.8005 (0.7917; 0.8084)	0.5323 (0.5003; 0.5569)	0.5000 (0.4949; 0.5036)
0.2,0.2; 0.4,0.4	2 x 0.2	2000	0.8164 (0.8048; 0.8255)	0.5517 (0.5364; 0.5741)	0.5000 (0.4963; 0.5024)
0.2,0.2; 0.4,0.4	2 x 0.2	10000	0.8174 (0.8140; 0.8213)	0.6909 (0.6706; 0.7253)	0.5000 (0.4989; 0.5028)
0.4,0.4; 0.4,0.4	2 x 0.05	200	0.4941 (0.4540; 0.5431)	0.4722 (0.4321; 0.5202)	0.5000 (0.4787; 0.5051)
0.4,0.4; 0.4,0.4	2 x 0.05	1000	0.5850 (0.5344; 0.6367)	0.5077 (0.4889; 0.5258)	0.5000 (0.4963; 0.5075)
0.4,0.4; 0.4,0.4	2 x 0.05	2000	0.6585 (0.6345; 0.6714)	0.5061 (0.4963; 0.5147)	0.5000 (0.4959; 0.5005)
0.4,0.4; 0.4,0.4	2 x 0.05	10000	0.6675 (0.6629; 0.6719)	0.5341 (0.5253; 0.5438)	0.5000 (0.4989; 0.5000)
0.4,0.4; 0.4,0.4	2 x 0.1	200	0.5147 (0.4555; 0.5712)	0.4624 (0.4277; 0.5220)	0.5000 (0.4857; 0.5000)
0.4,0.4; 0.4,0.4	2 x 0.1	1000	0.7054 (0.6815; 0.7273)	0.5104 (0.4918; 0.5328)	0.5000 (0.4993; 0.5065)
0.4,0.4; 0.4,0.4	2 x 0.1	2000	0.7302 (0.7191; 0.7403)	0.5157 (0.5024; 0.5292)	0.5000 (0.4949; 0.5007)
0.4,0.4; 0.4,0.4	2 x 0.1	10000	0.7332 (0.7290; 0.7391)	0.5750 (0.5604; 0.6000)	0.5000 (0.5000; 0.5008)
0.4,0.4; 0.4,0.4	2 x 0.2	200	0.5200 (0.4829; 0.6178)	0.4764 (0.4308; 0.5242)	0.5000 (0.4765; 0.5000)
0.4,0.4; 0.4,0.4	2 x 0.2	1000	0.8051 (0.7879; 0.8191)	0.5261 (0.5002; 0.5464)	0.5000 (0.4962; 0.5042)
0.4,0.4; 0.4,0.4	2 x 0.2	2000	0.8125 (0.8035; 0.8229)	0.5431 (0.5315; 0.5696)	0.5000 (0.4959; 0.5005)
0.4,0.4; 0.4,0.4	2 x 0.2	10000	0.8151 (0.8111; 0.8184)	0.6761 (0.6345; 0.7042)	0.5000 (0.4999; 0.5004)

Performance of the algorithms `MBMDRC`, `RANGER`, and `GLMNET` measured as AUC over 50 replicates in scenario 5. The median of the AUC and the 25% and 75% quantile in parentheses over 50 replicates are given.
