# Supplementary material for: Empowering individual trait prediction using interactions for precision medicine
Source: BMC Bioinformatics. 2021 Feb 18;22:74. doi: 10.1186/s12859-021-04011-z (PMC7890638; doi:10.1186/s12859-021-04011-z)
Supplement: Supplementary file 10 — Additional file 10: Table 7. Performance in scenario 7. Performance of the algorithms MBMDRC, RANGER, and GLMNET measured as AUC over 50 replicates in scenario 7: three pairs of interacting SNPs without marginal effects and three SNPs with marginal effects only (MAF 0.1, 0.2, or 0.4 and heritability 0.05, 0.1, 0.2), 91 SNPs without any effect.. The median of the AUC and the 25% and 75% quantile in parentheses over 50 replicates are given. [file 12859_2021_4011_MOESM10_ESM.pdf]

Table 14 Performance in scenario 7.

MAF	h^2	n	MBMDRC	RANGER	GLMNET
0.1,0.1; 0.2,0.2; 0.4,0.4; 0.1; 0.2; 0.4	6 x 0.05	200	0.5398 (0.4819; 0.5874)	0.5316 (0.5021; 0.5890)	0.5282 (0.5000; 0.5956)
0.1,0.1; 0.2,0.2; 0.4,0.4; 0.1; 0.2; 0.4	6 x 0.05	1000	0.6594 (0.6263; 0.6825)	0.6523 (0.6358; 0.6710)	0.6596 (0.6374; 0.6772)
0.1,0.1; 0.2,0.2; 0.4,0.4; 0.1; 0.2; 0.4	6 x 0.05	2000	0.7407 (0.7150; 0.7602)	0.6688 (0.6521; 0.6817)	0.6687 (0.6511; 0.6821)
0.1,0.1; 0.2,0.2; 0.4,0.4; 0.1; 0.2; 0.4	6 x 0.05	10000	0.7707 (0.7650; 0.7781)	0.6938 (0.6882; 0.7093)	0.6811 (0.6704; 0.6871)
0.1,0.1; 0.2,0.2; 0.4,0.4; 0.1; 0.2; 0.4	6 x 0.1	200	0.6009 (0.5340; 0.6414)	0.6114 (0.5833; 0.6748)	0.6176 (0.5736; 0.6615)
0.1,0.1; 0.2,0.2; 0.4,0.4; 0.1; 0.2; 0.4	6 x 0.1	1000	0.8087 (0.7657; 0.8445)	0.7167 (0.6968; 0.7320)	0.7109 (0.6912; 0.7243)
0.1,0.1; 0.2,0.2; 0.4,0.4; 0.1; 0.2; 0.4	6 x 0.1	2000	0.8533 (0.8385; 0.8697)	0.7326 (0.7219; 0.7475)	0.7213 (0.7078; 0.7441)
0.1,0.1; 0.2,0.2; 0.4,0.4; 0.1; 0.2; 0.4	6 x 0.1	10000	0.8639 (0.8554; 0.8737)	0.7732 (0.7631; 0.7838)	0.7360 (0.7283; 0.7465)
0.1,0.1; 0.2,0.2; 0.4,0.4; 0.1; 0.2; 0.4	6 x 0.2	200	0.6080 (0.5592; 0.6636)	0.6376 (0.5958; 0.7031)	0.6282 (0.5608; 0.6964)
0.1,0.1; 0.2,0.2; 0.4,0.4; 0.1; 0.2; 0.4	6 x 0.2	1000	0.8861 (0.8577; 0.9070)	0.7510 (0.7328; 0.7710)	0.7374 (0.7208; 0.7526)
0.1,0.1; 0.2,0.2; 0.4,0.4; 0.1; 0.2; 0.4	6 x 0.2	2000	0.9101 (0.9025; 0.9197)	0.7858 (0.7706; 0.7996)	0.7491 (0.7332; 0.7616)
0.1,0.1; 0.2,0.2; 0.4,0.4; 0.1; 0.2; 0.4	6 x 0.2	10000	0.9231 (0.9165; 0.9256)	0.8387 (0.8323; 0.8457)	0.7608 (0.7522; 0.7651)

Performance of the algorithms `MBMDRC`, `RANGER`, and `GLMNET` measured as AUC over 50 replicates in scenario 7. The median of the AUC and the 25% and 75% quantile in parentheses over 50 replicates are given.
