# Supplementary material for: Empowering individual trait prediction using interactions for precision medicine
Source: BMC Bioinformatics. 2021 Feb 18;22:74. doi: 10.1186/s12859-021-04011-z (PMC7890638; doi:10.1186/s12859-021-04011-z)
Supplement: Supplementary file 12 — Additional file 12: Statistical background. Detailed information on the statistical background and the connection between regression model coefficients and penetrance tables. [file 12859_2021_4011_MOESM12_ESM.docx]

Empowering individual trait prediction using interactions for precision medicine

**Damian Gola**
Institut für Medizinische Biometrie und Statistik
Universität zu Lübeck
Universitätsklinikum Schleswig-Holstein, Campus Lübeck
Lübeck, Germany
[gola@imbs.uni-luebeck.de](mailto:gola@imbs.uni-luebeck.de)

**Inke R. König***
Institut für Medizinische Biometrie und Statistik
Universität zu Lübeck
Universitätsklinikum Schleswig-Holstein, Campus Lübeck
Lübeck, Germany
[inke.koenig@imbs.uni-luebeck.de](mailto:inke.koenig@imbs.uni-luebeck.de)

* Corresponding author

# Statistical Background

## Statistical interactions

In statistics, two variables are said to be statistically independent if the influence of them on the target size can be modeled as a function with two separate parameters for the two variables. If an additional parameter is needed to describe the effect of both variables together on the target variable, this is referred to as interaction between the two variables in statistics. The presence of a statistical interaction depends on the type of model used for modelling. In the following, we will explain this in the light of a classification task in genetic epidemiology, i.e. the prediction of a dichotomous outcome $Y$ based on genetic markers, here given as single nucleotide polymorphisms (SNPs) with two alleles $A_{1}$ and $A_{2}$.

### Definition via logistic regression

Cordell [1] defines the interaction of two SNPs $A$ and $B$ with alleles $A_{1}$, $A_{2}$ and $B_{1}$, $B_{2}$ on the basis of a saturated linear model, given by

$$\begin{matrix} l\left( \mathbb{E}\left( Y \right) \right)= & \beta_{0}+\beta_{A_{1}A_{2}}^{A}\mathbb{1}_{A_{1}A_{2}}+\beta_{A_{2}A_{2}}^{A}\mathbb{1}_{A_{1}A_{2}}+\beta_{B_{1}B_{2}}^{B}\mathbb{1}_{B_{1}B_{2}}+\beta_{B_{1}B_{2}}^{B}\mathbb{1}_{B_{1}B_{2}}+ \\ & \iota_{A_{1}A_{2}B_{1}B_{2}}\mathbb{1}_{A_{1}A_{2}B_{1}B_{2}}+\iota_{A_{1}A_{2}B_{2}B_{2}}\mathbb{1}_{A_{1}A_{2}B_{2}B_{2}}+ \\ & \iota_{A_{2}A_{2}B_{1}B_{2}}\mathbb{1}_{A_{2}A_{2}B_{1}B_{2}}+\iota_{A_{2}A_{2}B_{2}B_{2}}\mathbb{1}_{A_{2}A_{2}B_{2}B_{2}} \end{matrix}.$$

Here, $l$ is a link function and, for a dichotomous outcome $Y$ which follows a Bernoulli distribution with parameter $\pi$, $l$ is the logarithm of the odds of $\pi=\mathbb{P}\left( Y=1 \right)$:

$$l\left( \mathbb{E}\left( Y \right) \right)=\mathrm{logit}\left( \pi\right)=\ln\left( \frac{\mathbb{P}\left( Y=1 \right)}{1-\mathbb{P}\left( Y=1 \right)} \right),$$

$\beta_{A_{1}A_{2}}^{A}$, $\beta_{A_{2}A_{2}}^{A}$, $\beta_{B_{1}B_{2}}^{B}$, $\beta_{B_{2}B_{2}}^{B}$ are the main effects , and $\iota_{A_{1}A_{2}B_{1}B_{2}}$, $\iota_{A_{1}A_{2}B_{2}B_{2}}$, $\iota_{A_{2}A_{2}B_{1}B_{2}}$, $\iota_{A_{2}A_{2}B_{2}B_{2}}$ are the interaction effects. The SNPs $A$ and $B$ are said to be interacting if at least one of the interaction effects is not equal to zero, i.e. there is deviation from additivity of the main effects.

To simplify the model, one can assume a recessive, dominant or additive genetic model for the SNPs. In this case, the regression model simplifies to

$$\mathrm{logit}\left( \pi\right)=\beta_{0}+\beta^{A}x^{A}+\beta^{B}x^{B}+\iota x^{A}x^{B},$$

where

$$x^{A}=\left\{ \begin{matrix} 1 A_{2}A_{2}, \\ 0 \mathrm{otherwise} \end{matrix} \right.$$

for a recessive genetic model,

$$x^{A}=\left\{ \begin{matrix} 0 A_{1}A_{1}, \\ 1 \mathrm{otherwise} \end{matrix} \right.$$

for a dominant genetic model and

$$x^{A}=\left\{ \begin{matrix} 0 A_{1}A_{1}, \\ 1 A_{1}A_{2}, \\ 2 A_{2}A_{2} \end{matrix} \right.$$

for an additive model for SNP $A$. The definitions for SNP $B$ are equivalent.

### Definition via penetrances

Table 1 Penetrance table. Example of a penetrance table for a multilocus $\mathcal{L=}\left( L_{1},L_{2} \right)$ with two diallelic SNPs $L_{1}=A$ and $L_{2}=B$.

|  |  | **Genotype at SNP** $B$ | | |
| --- | --- | --- | --- | --- |
|  |  | $B_{1}B_{1}$ | $B_{1}B_{2}$ | $B_{2}B_{2}$ |
| **Genotype at SNP** $A$ | $A_{1}A_{1}$ | 0.62 | 0.62 | 0.82 |
|  | $A_{1}A_{2}$ | 0.62 | 0.62 | 0.82 |
|  | $A_{2}A_{2}$ | 0.73 | 0.73 | 0.88 |

Figure 1 Penetrances. Visualization of penetrances of a multilocus $\mathcal{L=}\left( L_{1},L_{2} \right)$ with two diallelic SNPs $L_{1}=A$ and $L_{2}=B$ as in Table 1.

The definition of statistical interaction is not limited to the deviation from the additivity of the main effects in the logistic model. Another definition is based on the penetrance, which corresponds to modelling of the probability $\pi=\mathbb{P}\left( Y=1 \right)$ by a linear model. The penetrance function is defined as

$$f^{A}\left( g \right)=\mathbb{P}\left( Y=1\mid G=g \right),$$

i.e. the probability of $Y=1$ given a specific genotype $g\in\left\{ A_{1}A_{1},A_{1}A_{2},A_{2}A_{2} \right\}=\left\{ 0,1,2 \right\}$ of SNP $A$. If multiple SNPs $L_{j}$, $j=1,\ldots,d$, $d\in\mathbb{N}^{+}$ as multilocus $\mathcal{L}=\left( L_{1},\ldots,L_{d} \right)$ are considered, the definition of the penetrance function can easily be extended to

$$f_{\mathbf{g}}^{\mathcal{L}}:=f^{\mathcal{L}}\left( g_{1},\ldots,g_{d} \right)=\mathbb{P}\left( Y=1\mid G_{1}=g_{1},\ldots,G_{d}=g_{d} \right).$$

Here, $g_{j}$ is one of three possible genotypes of SNP $L_{j}$ and $\mathbf{g}=g_{1},\ldots,g_{d}$ is one specific combination of genotypes. Similar to the definition in the regression framework, an interaction between loci in terms of penetrance may be expressed as deviation from the additivity in the penetrance changes per allele. As an example, assume two SNPs $A$ and $B$ with alleles $A_{1}$, $A_{2}$ and $B_{1}$, $B_{2}$. Further, assume that at SNP $A$ the penetracne increases from $f^{A}\left( 0 \right)=f^{A}\left( 1 \right)=0.62$ by 0.11 to $f^{A}\left( 2 \right)=0.73$ for genotype $A_{2}A_{2}$ compared to the genotypes $A_{1}A_{1}$ and $A_{1}A_{2}$. At the same time, the penetrance is increased from $f^{B}\left( 0 \right)=f^{B}\left( 1 \right)=0.62$ for the genotypes $B_{1}B_{1}$ and $B_{1}B_{2}$ by 0.2 to $f^{B}\left( 2 \right)=0.82$ if the genotype $B_{2}B_{2}$ is present at SNP B. If both genotypes $A_{2}A_{2}$ and $B_{2}B_{2}$ are present at the same time, a penetrance of $f_{2,2}^{A,B}=0.62+0.11+0.2=0.93$ would be expected. However, in this example $f_{2,2}^{A,B}=0.88$ (see Table 1 and Figure 1). Thus, there is a deviation of $-0.05$ from additivity and therefore the SNPs $A$ and $B$ could be considered as interacting.

Table 2 Penetrance table without any marginal effects. Example of a penetrance table for a multilocus $\mathcal{L=}\left( L_{1},L_{2} \right)$ with two diallelic SNPs $L_{1}=A$ and $L_{2}=B$ without any marginal effects.

|  |  | **Genotype at SNP** $B$ | | | **Marginal penetrance with respect to** $B$ |
| --- | --- | --- | --- | --- | --- |
|  |  | $B_{1}B_{1}$ (0.25) | $B_{1}B_{2}$ (0.5) | $B_{2}B_{2}$ (0.25) |  |
| **Genotype at SNP** $A$ | $A_{1}A_{1}$ (0.25) | 0 | 0 | 1 | 0.25 |
|  | $A_{1}A_{2}$ (0.5) | 0 | 0.5 | 0 | 0.25 |
|  | $A_{2}A_{2}$ (0.25) | 1 | 0 | 0 | 0.25 |
| **Marginal penetrance with respect to** $A$ |  | 0.25 | 0.25 | 0.25 |  |

If a SNP $L_{j}$ falls outside the consideration of the multilocus $\mathcal{L}$, then the penetrances of the resulting multilocus genotypes are

$$f_{g_{1},\ldots,g_{j-1},g_{j+1},\ldots,g_{d}}^{\mathcal{L}\backslash L_{j}}=\sum_{g_{j}} \mathbb{P}\left( G_{j}=g_{j} \right)f_{g_{1},\ldots,g_{j-1},g_{j},g_{j+1},\ldots,g_{d}}^{\mathcal{L}}.$$

We refer to $f_{g_{1},\ldots,g_{j-1},g_{j+1},\ldots,g_{d}}^{\mathcal{L}\backslash L_{j}}$ as marginal penetrances with respect to $L_{j}$. If

$$f_{g_{1},\ldots,g_{j-1},g_{j+1},\ldots,g_{d}}^{\mathcal{L}\backslash L_{j}}=f^{\mathcal{L}\backslash L_{j}} \forall g_{1},\ldots,g_{j-1},g_{j+1},\ldots,g_{d},$$

then we say that $L_{j}$ has no marginal effect. An example of this concept is given in Table 2 for a multilocus $\mathcal{L}$ with two diallelic SNPs. The respective genotype probabilities are given in parentheses. If SNP $A$ is ignored, the penetrance on each genotype of SNP $B$ is 0.25. Conversely, the penetrances of SNP $A$ are also 0.25 if SNP $B$ is ignored. Thus, both SNPs have no marginal effect. In this example, it is even the case that the marginal penetrances are identical with respect to both SNPs. If these loci were analyzed independently, no effect would be identifiable.

Using penetrances two measures can be calculated, which give information about the influence of the considered multilocus $\mathcal{L}$ on an outcome $Y$. For this purpose Urbanowicz et al. [2] calculate the population prevalence $K$ as

$$K=\sum_{\mathbf{g}\in\mathbf{G}} \mathbb{P}\left( \mathbf{g} \right)f_{\mathbf{g}}^{\mathcal{L}}.$$

This expresses the probability with which a phenotype can be observed in a population. Based on $K$, the heritability

$$h^{2}=\frac{1}{K\left( 1-K \right)}\sum_{\mathbf{g}\in\mathbf{G}} \mathbb{P}\left( \mathbf{g} \right)\left( f_{\mathbf{g}}^{\mathcal{L}}-K \right)^{2}$$

can also be calculated for $\mathcal{L}$ as a measure of the explained variance of $Y$ by $\mathcal{L}$. The larger $h^{2}\in[0,1]$, the better $Y$ can be explained only by considering $\mathcal{L}$.

### Connection between logistic regression and penetrance

Table 3 Log-odds of penetrances in Table 2 for two diallelic SNPs $A$ and $B$.

|  |  |  | **Genotype at SNP** $B$ |  |
| --- | --- | --- | --- | --- |
|  |  | $B_{1}B_{1}$ | $B_{1}B_{2}$ | $B_{2}B_{2}$ |
|  | $A_{1}A_{1}$ | 0.5 | 0.5 | 1.5 |
| **Genotype at SNP** $A$ | $A_{1}A_{2}$ | 0.5 | 0.5 | 1.5 |
|  | $A_{2}A_{2}$ | 1 | 1 | 2 |

In order to support the description of the simulation setting, we give a short explanation on how to convert the effects on a logistic regression scale to penetrances and vice versa.

The logarithmic odds at the multilocus $\mathcal{L}$ can be calculated by

$$\begin{matrix} \mathrm{logit}\left( f_{\mathbf{g}}^{\mathcal{L}} \right)=\beta_{0}+\sum_{k=1}^{i} \sum_{\mathbf{G}_{k}} \iota_{\mathbf{g}_{k}} \end{matrix},$$

given effect parameters $\iota$. In this notation, $\mathbf{G}_{k}$ stands for all $k$ tuples of the genotype combinations of $\mathbf{g}$, and $0\leq i\leq d$ is the number of genotypes in $\mathbf{g}$ different from the homozygous genotype with the most common allele at SNP $L_{j}$, $j=1,\ldots,d$. In particular, for $k=1$, the $\iota_{\mathbf{g}_{1}}$ are the corresponding main effects $\beta_{g_{j}}$, $g_{j}\in\mathbf{G}_{1}$.

With the inverse of the $\mathrm{logit}$ function $\mathrm{logit}^{-1}\left( z \right)=\mathrm{expit}\left( z \right):=\frac{\exp\left( z \right)}{1+\exp\left( z \right)}$ the penetrance for each genotype combination can be calculated from the effect parameters of the logistic model.

Conversely, from a given penetrance $f_{\mathbf{g}}^{\mathcal{L}}$ for all genotype combinations $\mathbf{g}$, the effect parameters $\iota_{\mathbf{g}}$ of the corresponding logistic model can be calculated by

$$\begin{matrix} \iota_{\mathbf{g}} & =\left( -1 \right)^{j}\tilde{f}_{\mathbf{0}}^{\mathcal{L}}+\left( -1 \right)^{j+1}\sum\tilde{f}_{\mathbf{G}_{1}}^{\mathcal{L}}+\ldots+\left( -1 \right)^{2j}\sum\tilde{f}_{\mathbf{G}_{j}}^{\mathcal{L}} \\ & =\left( -1 \right)^{j}\tilde{f}_{\mathbf{0}}^{\mathcal{L}}+\sum_{k=1}^{j} \left( \left( -1 \right)^{j+k}\sum\tilde{f}_{\mathbf{G}_{k}}^{\mathcal{L}} \right) \end{matrix}.$$

Here, $\tilde{f}_{\mathbf{g}}^{\mathcal{L}}=\mathrm{logit}\left( f_{\mathbf{g}}^{\mathcal{L}} \right)$ and $f_{\mathbf{0}}$ is the penetrance of the genotype combination of the homozygous genotypes with the most common allele at the respective SNP.

It should be noted at this point that an interaction effect at one level does not automatically imply an interaction effect at the other level. This is called a scale effect [3] and discussed in detail by Gola et al. [4]. As an example, Table 3 shows the combinations of penetrances transformed into effect parameters of the logistic regression model from Table 2. It can be seen that there is no deviation from additivity at the level of logistic regression. The logarithmic odds increase for the genotype $A_{2}A_{2}$ at SNP A by $\beta^{A}=0.5$ and for the genotype $B_{2}B_{2}$ at SNP B by $\beta^{B}=1$. When combining both genotypes, the logarithmic odds increase by $\beta^{A}+\beta^{B}+\iota=0.5+1+0=1.5$.

This effect may also lead to the effect that a pure interaction of SNPs without any main or marginal effects of the single SNPs on one level being an impure interaction of SNPs with main or marginal effects on the other level and thus easier to detect by appropriate methods.

# Selected hyperparameters

Inspection of the hyperparameters selected by tuning for the MBMDRC models shows that they correspond to the respective scenarios, i.e. the scenario settings determine the optimal hyperparameter settings. That, in turn, enables to select hyperparameter settings to include certain characteristics in prediction.

Table 1 Selected hyperparameters by tuning in scenario 1.

| order_range | order | adjustment | Selection frequency (%) | alpha | min_cell_size |
| --- | --- | --- | --- | --- | --- |
| FALSE | 1 | CODOMINANT | 13.04 | 0.2744 (0.0950, 0.6319) | 26 (11, 38) |
| FALSE | 1 | NONE | 4.44 | 0.2631 (0.0771, 0.6532) | 25 (8, 42) |
| FALSE | 2 | CODOMINANT | 3.65 | 0.5040 (0.2370, 0.7539) | 23 (14, 43) |
| FALSE | 2 | NONE | 29.48 | 0.4472 (0.1883, 0.7452) | 18 (7, 34) |
| TRUE | 1 | CODOMINANT | 6.26 | 0.2199 (0.0590, 0.5655) | 23 (10, 39) |
| TRUE | 1 | NONE | 3.39 | 0.2694 (0.0816, 0.6624) | 27 (8, 40) |
| TRUE | 2 | CODOMINANT | 17.83 | 0.4257 (0.1727, 0.7420) | 25 (13, 38) |
| TRUE | 2 | NONE | 21.91 | 0.4382 (0.1663, 0.7345) | 20 (8, 37) |

For hyperparameters alpha and min_cell_size the median and the first and third quartile in parentheses are given.

Table 2 Selected hyperparameters by tuning in scenario 3.

| order_range | order | adjustment | Selection frequency (%) | alpha | min_cell_size |
| --- | --- | --- | --- | --- | --- |
| FALSE | 1 | CODOMINANT | 2.67 | 0.3112 (0.0986, 0.6948) | 29 (15, 45) |
| FALSE | 1 | NONE | 1.37 | 0.3781 (0.1633, 0.7290) | 28 (14, 46) |
| FALSE | 2 | CODOMINANT | 30.89 | 0.5152 (0.2113, 0.8135) | 13 (5, 29) |
| FALSE | 2 | NONE | 19.23 | 0.5267 (0.2331, 0.7976) | 12 (4, 28) |
| TRUE | 1 | CODOMINANT | 2.14 | 0.3411 (0.1078, 0.6796) | 29 (15, 45) |
| TRUE | 1 | NONE | 1.34 | 0.2683 (0.0915, 0.5463) | 30 (13, 45) |
| TRUE | 2 | CODOMINANT | 24.17 | 0.5132 (0.2033, 0.7986) | 12 (4, 27) |
| TRUE | 2 | NONE | 18.19 | 0.5055 (0.2098, 0.7961) | 14 (5, 30) |

For hyperparameters alpha and min_cell_size the median and the first and third quartile in parantheses are given.

Table 3 Selected hyperparameters by tuning in scenario 7.

| order_range | order | adjustment | Selection frequency (%) | alpha | min_cell_size |
| --- | --- | --- | --- | --- | --- |
| FALSE | 1 | CODOMINANT | 4.67 | 0.5347 (0.1374, 0.7596) | 20 (10, 36) |
| FALSE | 1 | NONE | 2.61 | 0.3341 (0.0806, 0.5735) | 14 (7, 30) |
| FALSE | 2 | CODOMINANT | 6.78 | 0.5620 (0.2932, 0.7883) | 18 (5, 38) |
| FALSE | 2 | NONE | 6.44 | 0.4080 (0.1837, 0.6850) | 14 (5, 23) |
| TRUE | 1 | CODOMINANT | 3.50 | 0.2639 (0.0942, 0.5165) | 15 (8, 29) |
| TRUE | 1 | NONE | 2.17 | 0.3347 (0.1032, 0.6110) | 22 (12, 34) |
| TRUE | 2 | CODOMINANT | 67.83 | 0.3983 (0.1705, 0.6853) | 16 (7, 31) |
| TRUE | 2 | NONE | 6.00 | 0.5597 (0.2392, 0.8168) | 16 (5, 30) |

For hyperparameters alpha and min_cell_size the median and the first and third quartile in parantheses are given.

For scenarios 1, 3, and 7, Tables 1, 2, and 3 summarize how often specific combinations of the hyperparameters order_range, order und adjustment were selected in tuning. These hyperparameters depend on each other, i.e. interaction effects of two SNPs without any marginal effects can enter the prediction model only if order is set to 2. Simultaneously, the hyperparameters order_range and adjustment influence whether additional marginal effects can be detected by the MB-MDR algorithm and thus used for prediction. Since setting adjustment to CODOMINANT will adjust for possible marginal effects, order_range should be set to TRUE, to allow for marginal effects. On the other hand, if order is set to 1, both adjustment and order_range become meaningless.

In scenario 1, order is set to 2 in 72.87% of the replicates, although no interactions are simulated in this scenario. However, if the hyperparameters order_range and order are set to FALSE and 2, respectively, the hyperparameter adjustment is set to NONE more often (29.48%) than to CODOMINANT (3.65%). Thus, the MB-MDR algorithms also account for marginal effects. This is not the case if order_range and order are set to TRUE and 2, respectively. In this setting, adjustment is almost equally frequent set to NONE (21.91%) and CODOMINANT (17.83%). The hyperparameter alpha seems to be dependent on the hyperparameter order. If order is set to 1 the median of alpha between 0.22 and 0.27 whereas the median when order set to 2 ranges higher between 0.43 and 0.50. However, the range of the quartiles is rather big, thus there seems to be no optimal value for alpha in general for this scenario. Same is true for the hyperparameter min_cell_size with a median between 18 and 27 and wide quartile ranges.

In scenario 3, order is set to 2 in 92.48% of the replicates, as expected, since in this scenario, only interaction effects are simulated. Although there are no marginal effects of single SNPs simulated in this scenario, there is a slight tendency to set adjustment to CODOMINANT, whereas the selection frequencies of the values TRUE (45.84%) and FALSE (54.16%) for the hyperparameter order_range are almost equal. Again, the hyperparameters alpha and min_cell_size depend on order and no optimal values can be determined because of the wide range of the quartiles.

In scenario 7 with simulated marginal and interaction effects, one combination of hyperparameters stands out. In this scenario the hyperparameters order, order_range, and adjustment are set to 2, TRUE, and CODOMINANT in 59.14% of the replicates, much more frequent than any other combination of hyperparameter values. Using these hyperparameter settings, marginal and interaction effects can clearly be differentiated and used for prediction. Again, no clear optimal values for the hyperparameters alpha and min_cell_size can be determined.

# References

1. Cordell HJ. Detecting gene-gene interactions that underlie human diseases. Nat Rev Genet. 2009;10:392–404. doi:[10.1038/nrg2579](https://doi.org/10.1038/nrg2579).

2. Urbanowicz RJ, Kiralis J, Sinnott-Armstrong N a, Heberling T, Fisher JM, Moore JH. GAMETES: a fast, direct algorithm for generating pure, strict, epistatic models with random architectures. BioData Min. 2012;5:16.

3. Kraft P. Statistical issues in epidemiological studies of gene-environment interaction. 2007. <https://hstalks.com/bs/79/>. Accessed 1 Nov 2018.

4. Gola D, Hessler N, Schwaninger M, Ziegler A, König IR. Evaluating predictive biomarkers for a binary outcome with linear versus logistic regression - Practical recommendations for the choice of the model. bioRxiv. 2018. doi:[10.1101/347096](https://doi.org/10.1101/347096).
