# Supplementary material for: Empowering individual trait prediction using interactions for precision medicine
Source: BMC Bioinformatics. 2021 Feb 18;22:74. doi: 10.1186/s12859-021-04011-z (PMC7890638; doi:10.1186/s12859-021-04011-z)
Supplement: Supplementary file 22 — Additional file 22: Illustration of data generation procedure. In scenario 4 one interaction of two SNPs and three single SNPs build up the underlying effect structure. In each replicate SNP data of an unlimited population is generated according to the MAF specifications, here 0.2 for L1 and L2, 0.1 for L3, 0.2 for L4, and 0.4 for L5. Penetrance tables are generated according to the scenario MAF and heritability specifications, i.e. h2 = 0.2. At each locus the penetrances according to the genotypes are added on the logit scale and transformed back to the probability scale using the expit function to create the total probability pTotal. The phenotype (case or control) is sampled from a Bernoulli distribution with success probability pTotal. From the population a random sample of cases and controls is drawn from the replication dataset D. [file 12859_2021_4011_MOESM22_ESM.pdf]

Scenario 4

one interaction of two SNPs ($L_1 \times L_2$) and three single SNPs (L_3, L_4, L_5) without interactions

Effect size: $h^2 = 0.2$ for $L_1 \times L_2$, L_3, L_4, and L_5

MAF: 0.2 for L_1 and L_2, 0.1 for L_3, 0.2 for L_4, and 0.4 for L_5

Replicate 1

Simulated Population SNP Data

L$_1$	L$_2$	L$_3$	L$_4$	L$_5$	L$_6$	···	L$_{100}$
0	0	0	0	0	1	···	0
1	0	0	1	2	1	···	1
2	1	0	1	0	1	···	1
0	0	0	0	1	1	···	0
0	0	0	0	0	0	···	0
⋮	⋮	⋮	⋮	⋮	⋮	···	⋮
0	1	1	0	1	1	···	1
0	0	1	1	0	1	···	2
0	2	1	0	0	0	···	1
0	0	0	1	2	0	···	2
⋮	⋮	⋮	⋮	⋮	⋮	···	⋮

Generated Penetrance Tables

$L_1 \times L_2$

		L$_2$		
	g	0	1	2
L$_1$	0	0.4213	0.0904	0.2469
	1	0.0995	0.7186	0.3704
	2	0.1743	0.5156	0.7973

L_3

		L$_2$
	g	0
L$_3$	0	0.2904
	1	0.8405
	2	0.9652

L_4

		L$_2$
	g	0
L$_3$	0	0.3663
	1	0.8128
	2	0.9496

L_5

		L$_2$
	g	0
L$_3$	0	0.4100
	1	0.7967
	2	0.9389

Penetrances per Sample and SNP Combination, and Simulated Phenotype

L$_1$ x L$_2$	L$_3$	L$_4$	L$_5$	Total	Y
0.4213	0.2904	0.3663	0.4100	0.107	0
0.0995	0.2904	0.8128	0.9389	0.751	1
0.5156	0.2904	0.8128	0.4100	0.568	1
0.4213	0.2904	0.3663	0.7967	0.403	1
0.4213	0.2904	0.3663	0.4100	0.107	0
⋮	⋮	⋮	⋮	⋮	⋮
0.0904	0.8405	0.3663	0.7967	0.543	1
0.4213	0.8405	0.8128	0.4100	0.920	0
0.2469	0.8405	0.3663	0.4100	0.410	1
0.4213	0.2904	0.8128	0.9389	0.952	1
⋮	⋮	⋮	⋮	⋮	⋮

p_{Total}: $\text{expit}\left(\text{logit}\left(L_1 \times L_2\right) + \text{logit}\left(L_3\right) + \text{logit}\left(L_4\right) + \text{logit}\left(L_5\right)\right)$

Y: Randomly drawn from a *Bernoulli* distribution with $\mathbb{P}\left(Y_i = 1\right) = p_{\text{Total}_i}$

Dataset D

ID	L$_1$	L$_2$	L$_3$	L$_4$	L$_5$	L$_6$	···	L$_{100}$	Y
1	0	0	0	0	0	1	···	0	0
2	0	0	0	0	0	0	···	0	0
3	0	0	1	1	0	1	···	2	0
4	1	0	0	0	0	1	···	0	0
5	0	0	1	0	0	0	···	0	0
	⋮	⋮	⋮	⋮	⋮	⋮	···	⋮	⋮
1996	0	1	1	0	1	1	···	1	1
1997	2	0	1	1	0	1	···	0	1
1998	0	2	1	0	0	0	···	1	1
1999	0	0	0	1	2	0	···	2	1
2000	0	1	0	0	0	2	···	0	1

...

Replicate 50

Simulated Population SNP Data

L$_1$	L$_2$	L$_3$	L$_4$	L$_5$	L$_6$	···	L$_{100}$
0	1	0	1	1	1	···	1
0	1	0	0	0	2	···	0
1	0	0	1	1	1	···	1
0	1	0	1	1	1	···	0
1	0	0	0	0	1	···	1
⋮	⋮	⋮	⋮	⋮	⋮	···	⋮
0	1	0	0	1	1	···	1
2	0	1	2	0	2	···	0
1	2	1	1	0	0	···	1
1	1	0	0	2	1	···	1
⋮	⋮	⋮	⋮	⋮	⋮	···	⋮

Generated Penetrance Tables

$L_1 \times L_2$

		L$_2$		
	g	0	1	2
L$_1$	0	0.5452	0.8811	0.7270
	1	0.8810	0.2308	0.5561
	2	0.7275	0.5552	0.4173

L_3

		L$_2$
	g	0
L$_3$	0	0.2904
	1	0.8405
	2	0.9652

L_4

		L$_2$
	g	0
L$_3$	0	0.3663
	1	0.8128
	2	0.9496

L_5

		L$_2$
	g	0
L$_3$	0	0.4100
	1	0.7967
	2	0.9389

Penetrances per Sample and SNP Combination, and Simulated Phenotype

L$_1$ x L$_2$	L$_3$	L$_4$	L$_5$	Total	Y
0.5452	0.2904	0.8128	0.7967	0.8930	0
0.5452	0.2904	0.3663	0.4100	0.1646	0
0.8810	0.2904	0.8128	0.7967	0.9810	1
0.8811	0.2904	0.8128	0.7967	0.9810	0
0.8810	0.2904	0.3663	0.4100	0.5489	1
⋮	⋮	⋮	⋮	⋮	⋮
0.8811	0.2904	0.3663	0.7967	0.8729	1
0.7275	0.8405	0.9496	0.4100	0.9946	0
0.5561	0.8405	0.8128	0.4100	0.9522	0
0.2308	0.2904	0.3663	0.9389	0.5217	1
⋮	⋮	⋮	⋮	⋮	⋮

p_{Total}: $\text{expit}\left(\text{logit}\left(L_1 \times L_2\right) + \text{logit}\left(L_3\right) + \text{logit}\left(L_4\right) + \text{logit}\left(L_5\right)\right)$

Y: Randomly drawn from a *Bernoulli* distribution with $\mathbb{P}\left(Y_i = 1\right) = p_{\text{Total}_i}$

Dataset D

ID	L$_1$	L$_2$	L$_3$	L$_4$	L$_5$	L$_6$	···	L$_{100}$	Y
1	0	1	0	1	1	1	···	1	0
2	0	1	0	0	0	2	···	0	0
4	0	1	0	1	1	1	···	0	0
6	0	0	0	0	0	1	···	0	0
10	0	2	0	1	0	0	···	0	0
	⋮	⋮	⋮	⋮	⋮	⋮	···	⋮	⋮
1996	0	1	1	0	1	1	···	1	1
1997	2	0	1	1	0	1	···	2	1
1998	0	0	1	0	0	0	···	1	1
1999	0	1	0	0	1	1	···	1	1
2000	1	1	0	0	2	1	···	1	1
